# Supplementary material for: Multimodal deep learning for Alzheimer’s disease dementia assessment
Source: Nat Commun. 2022 Jun 20;13:3404. doi: 10.1038/s41467-022-31037-5 (PMC9209452; doi:10.1038/s41467-022-31037-5)
Supplement: Supplementary file 1 — Supplementary Information [file 41467_2022_31037_MOESM1_ESM.pdf]

Multimodal deep learning for Alzheimer's disease dementia assessment

Supplementary Information

Supplementary Figure S1

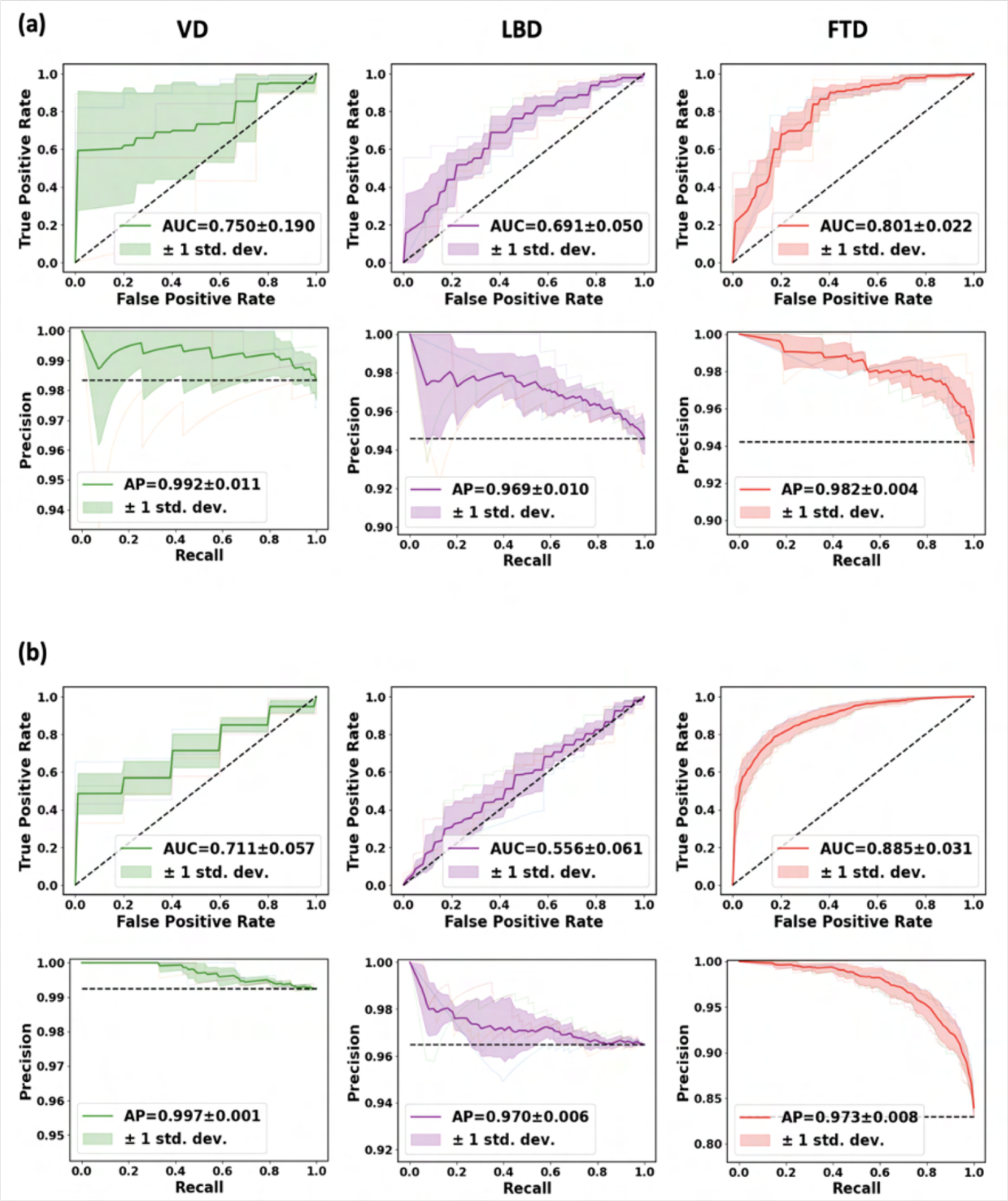

Supplementary Figure S1: MRI-only model performance on ADD task across nADD sub-groups. The ROC and PR curves demonstrate the ability of the MRI-only model to accurately delineate AD from nADD cases when evaluated across several nADD subgroups, including vascular dementia (VD), Lewy body dementia (LBD), and frontotemporal dementia (FTD). The area under ROC and PR curves demonstrate that model performance is the strongest on non-Parkinsonian dementias (VD and FTD) than Parkinsonian dementias. Performance curves for the (a) NACC test set and (b) all external datasets are shown.

Supplementary Figure S2

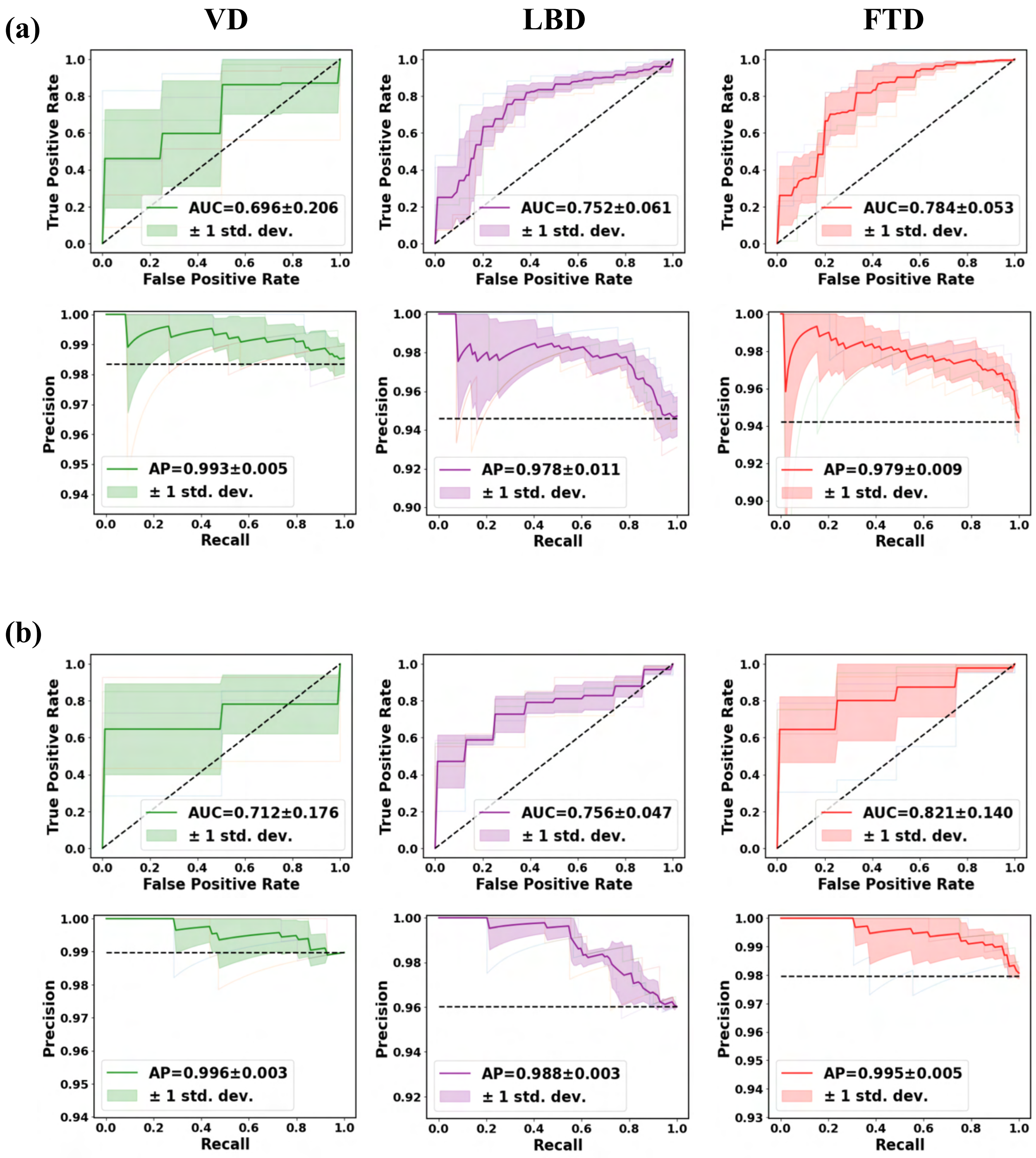

Supplementary Figure S2: Fusion model performance on ADD task across nADD sub-groups. The ROC and PR curves demonstrate the ability of the fusion (CNN + CatBoost) model to accurately delineate AD from nADD cases when evaluated across the nADD subgroups. The area under ROC and PR curves demonstrate that the inclusion of non-imaging data elevates the model's performance on Parkinsonian dementias. Performance curves for the (a) NACC test set and (b) OASIS dataset are shown.

Supplementary Figure S3

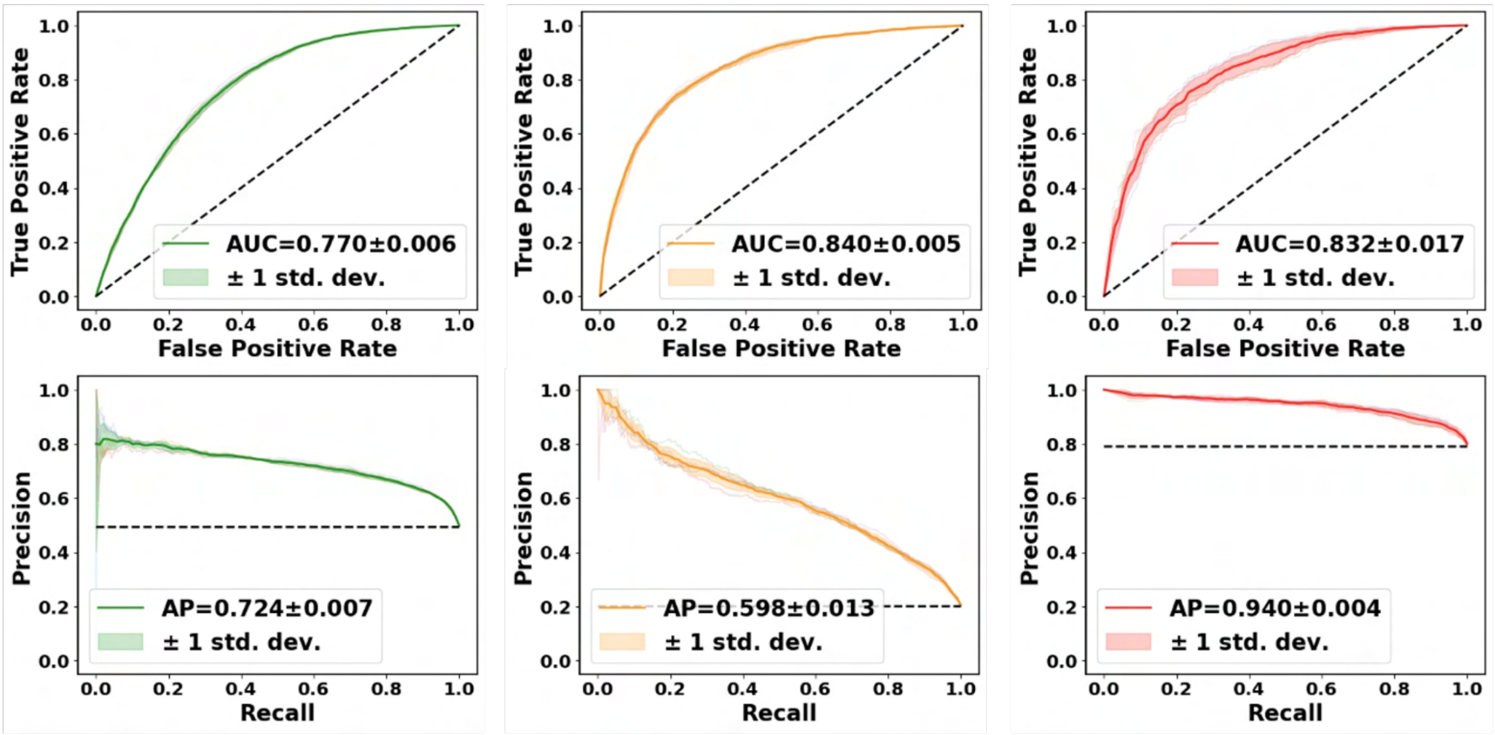

Supplementary Figure S3: MRI-only model ROC & PR curves on external datasets. The performance of the MRI-only CNN model on the combined external testing sets from seven cohorts is demonstrated. Note that the ROC and PR curves are demonstrated for the COG_{NC}(green), COG_{DE}(yellow), and ADD (red) tasks.

Supplementary Figure S4

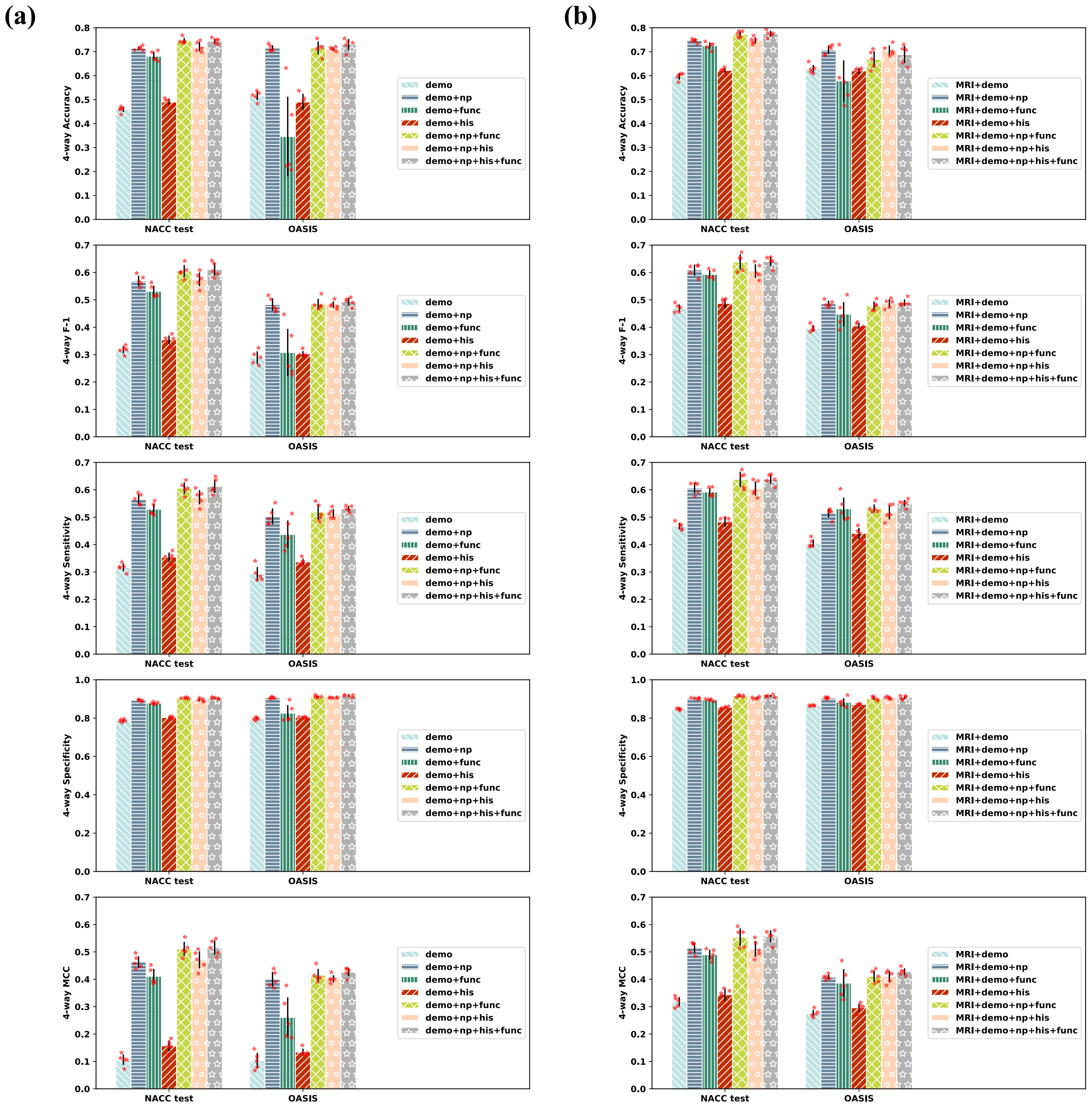

Supplementary Figure S4: Non-imaging and fusion models with partial features. We assessed the performance of 4-way classification on non-imaging and fusion (CNN + CatBoost) models when using varying combinations of historical ("his"), neuropsychological ("np"), and functional ("func") variables (as well as MRI-derived variables in the case of the fusion model). The panel (a) on the left shows the models' performance using different feature combinations but without MRI information. The panel (b) on the right shows the model performance using various feature combinations with MRI included. Error bars demonstrating mean +/- 1 standard deviation are derived on n=5 rounds of cross-validation. The model accuracy, F-1, sensitivity, specificity, and MCC values are demonstrated, and comparison is made between the NACC test set and the OASIS dataset for each performance metric. Of note, similar distributions of performance metrics are observed between the two datasets, thus suggesting that the model does not privilege particular features in one dataset over the other.

Supplementary Figure S5

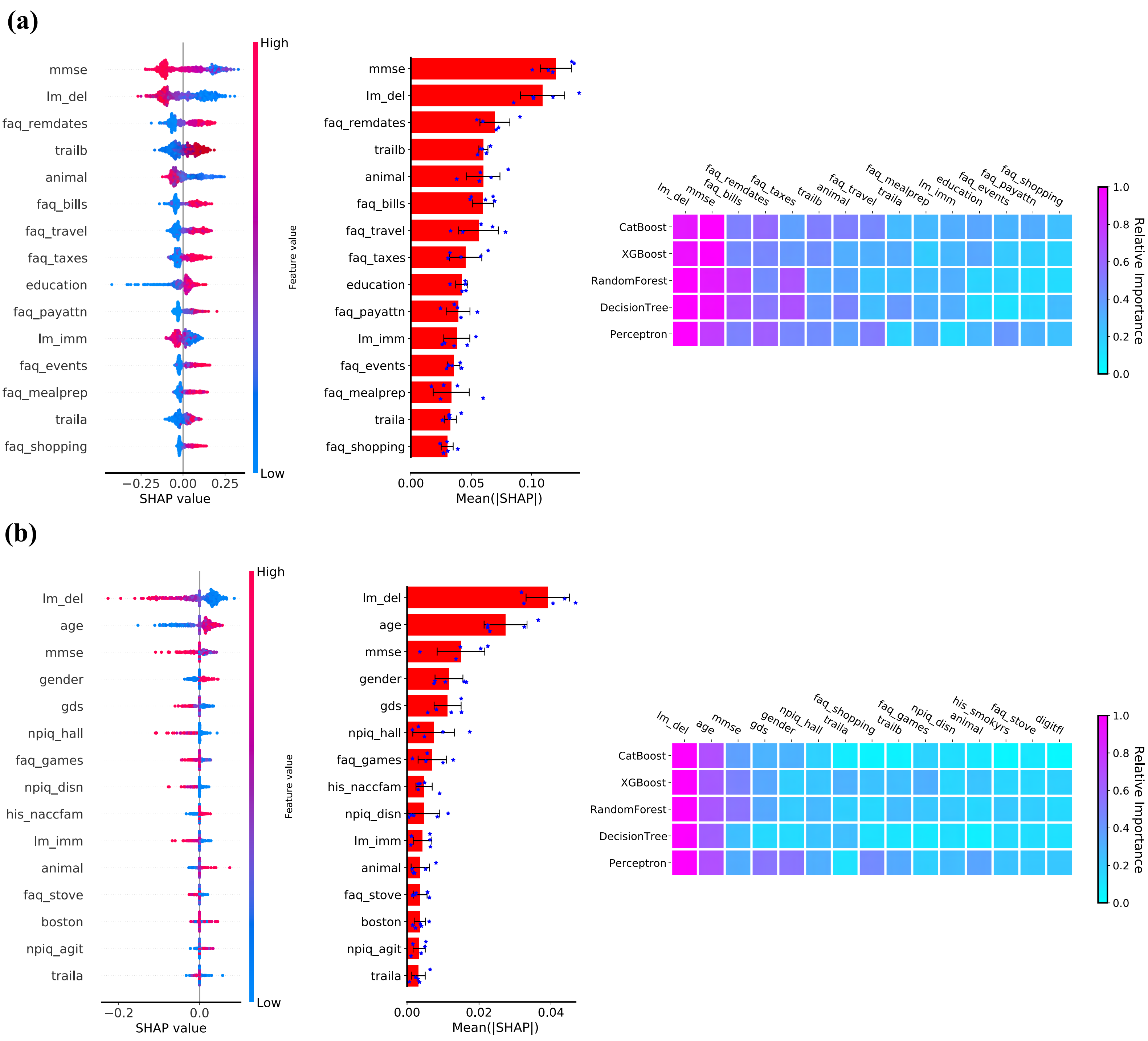

Supplementary Figure S5: SHAP analysis of non-imaging features. The importance of various historical, demographic, neuropsychological, and functional testing results for the (a) COG and (b) ADD task was assessed across five different machine learning models trained to make these predictions. Results here demonstrate feature importance without consideration of MRI-derived DEMO and ALZ scores. All results demonstrated herein are derived from the NACC test set, and error bars demonstrating mean +/- 1 standard deviation are derived on n =5 rounds of cross-validation. Of note, common hierarchies of feature importance emerged across classifiers when SHAP values are plotted in descending order as columns of a heatmap. Canonical predictors of dementia status and AD, including logical memory/delayed recall testing and MMSE are observed to display particularly high SHAP values across all classifiers.

Supplementary Figure S6

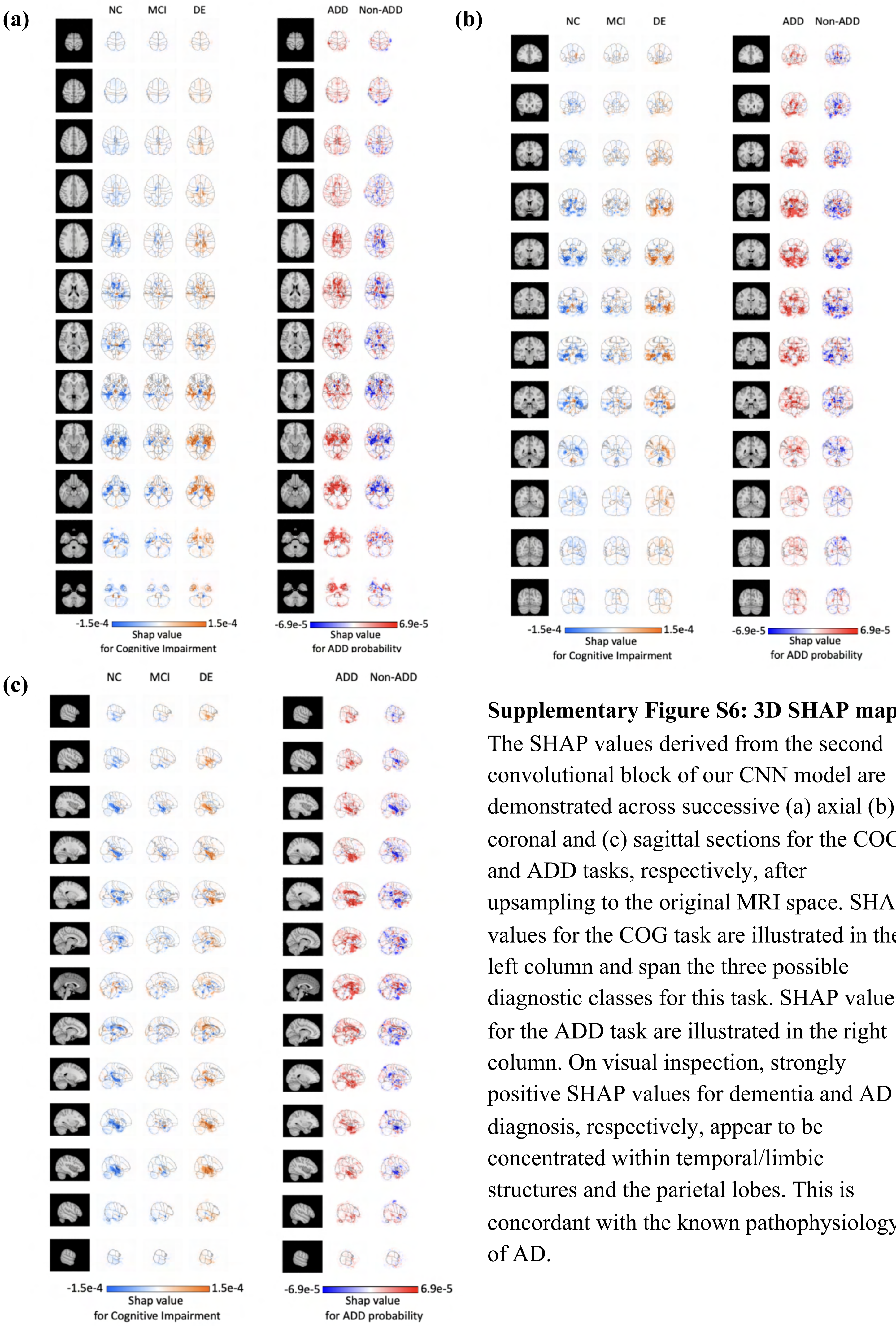

Supplementary Figure S6: 3D SHAP map. The SHAP values derived from the second convolutional block of our CNN model are demonstrated across successive (a) axial (b) coronal and (c) sagittal sections for the COG and ADD tasks, respectively, after upsampling to the original MRI space. SHAP values for the COG task are illustrated in the left column and span the three possible diagnostic classes for this task. SHAP values for the ADD task are illustrated in the right column. On visual inspection, strongly positive SHAP values for dementia and AD diagnosis, respectively, appear to be concentrated within temporal/limbic structures and the parietal lobes. This is concordant with the known pathophysiology of AD.

Supplementary Figure S7

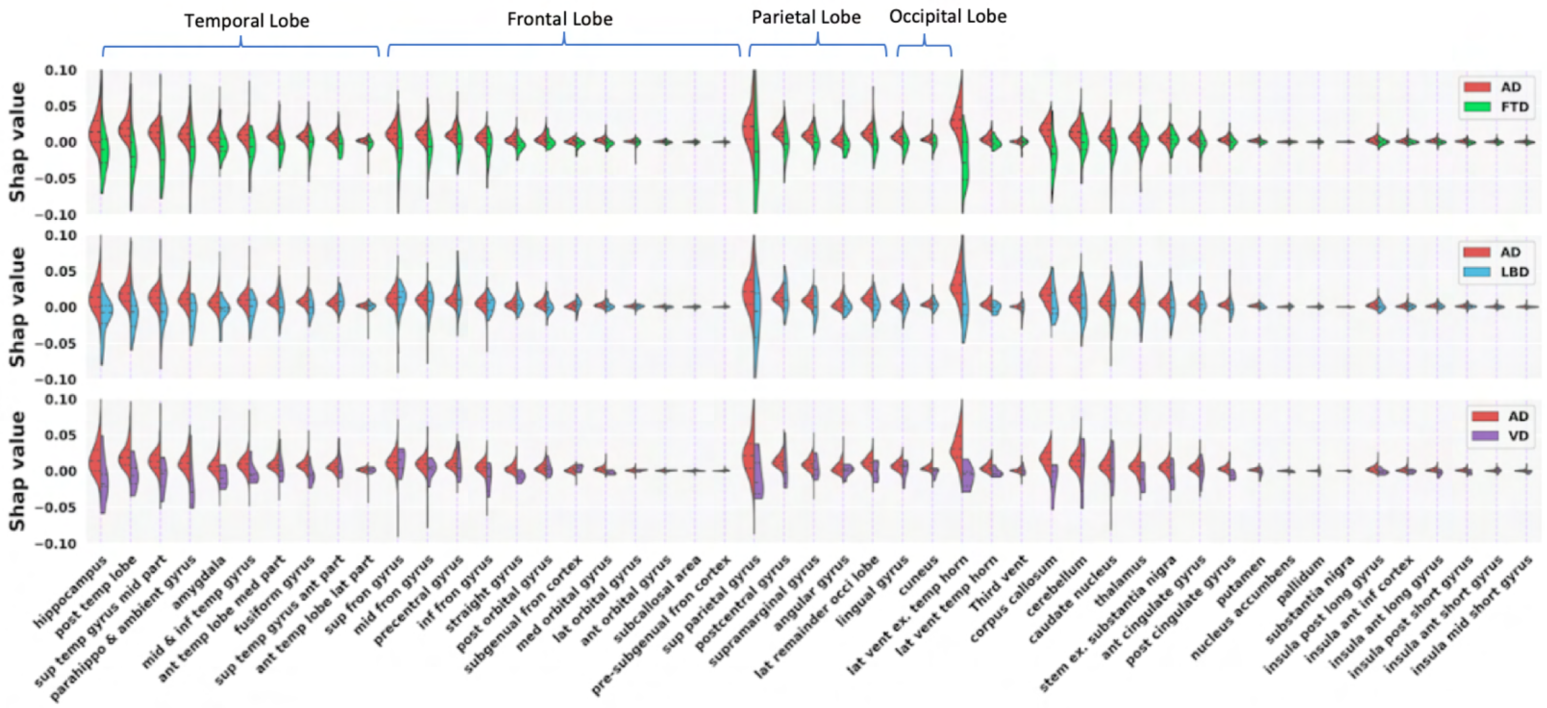

Supplementary Figure S7: SHAP based atrophy signature of non-AD dementias. We presented the distribution of the regionally-averaged SHAP values from the subjects that were correctly predicted as AD or non-AD dementias. The x-axis contains the region names that each violin plot is corresponding to. The regions were defined based on the Hammersmith Adult brain atlas. If there are same structures in both the left and right hemispheres, we merged them into one region, otherwise the regions from the altas were directly included in this plot. The order of the region names is the same as the **Fig. 4c** which was originally determined by ranking the mean absolute SHAP values descendingly within each lobe. The regionally-averaged SHAP value was derived by overlapping the segmentation mask of a region on the 3D shap heatmap generated using the DeepSHAP method of the MRI model on the NACC testing set. Comparisons on the regionally-averaged SHAP distribution were made between AD and each non-AD dementias, including (a) frontotemporal dementia (top row), (b) Lewy body dementia (middle row), and (c) vascular dementia (bottom row).

Supplementary Figure S8

A score	LSMEAN
0	0.9423999
1	1.1270171
2	1.0173903
3	1.2767164

B score	LSMEAN
0	0.9062537
1	0.9464061
2	1.2820319
3	1.422658

C score	LSMEAN
0	1.0185765
1	1.1609721
2	1.2777793
3	1.2811677

A score				
	0	1	2	3
0		0.1703	0.892	0.0003
1	0.1703		0.6144	0.0582
2	0.892	0.6144		0.0079
3	0.0003	0.0582	0.0079	

B score				
	0	1	2	3
0		0.9468	<.0001	<.0001
1	0.9468		<.0001	<.0001
2	<.0001	<.0001		0.0221
3	<.0001	<.0001	0.0221	

C score				
	0	1	2	3
0		0.1804	<.0001	<.0001
1	0.1804		0.3700	0.2606
2	<.0001	0.3700		0.9999
3	<.0001	0.2606	0.9999	

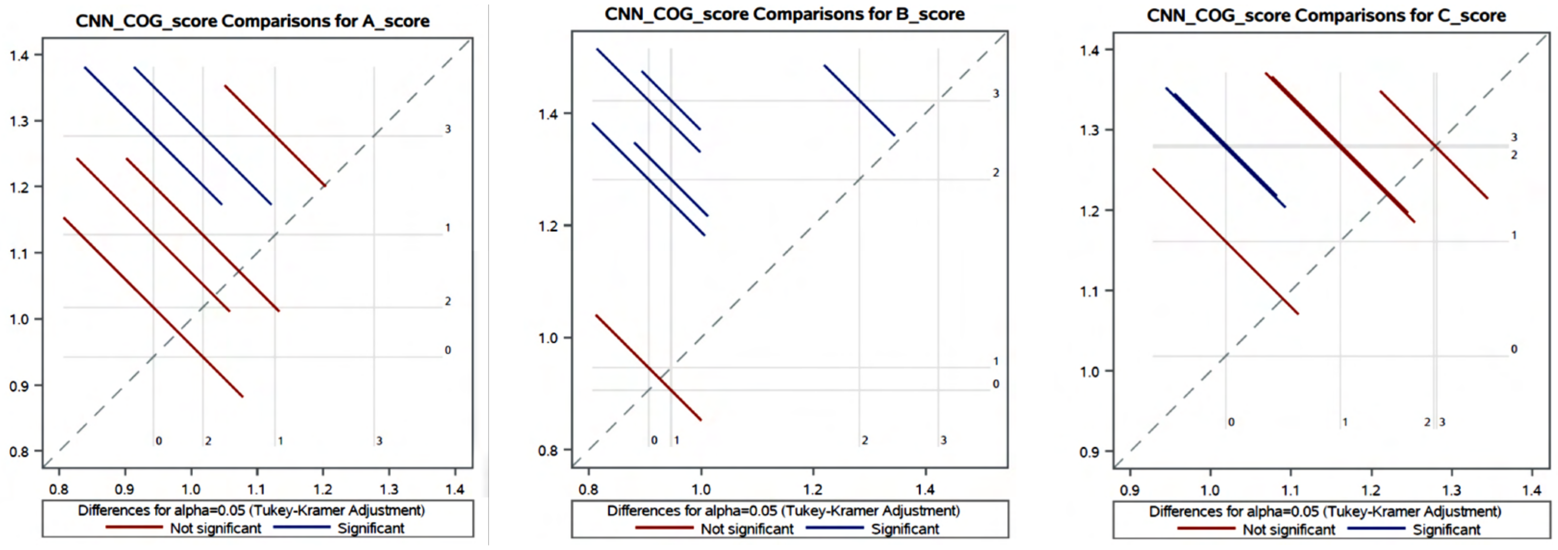

Supplementary Figure S8: Pairwise Tukey range test results for neuropathology.
To supplement the ANOVA analysis (Fig. 5c), we used the Tukey-Kramer test to assess mean pairwise differences in the CNN-derived DEMO scores on persons stratified by severity of neuropathologic findings. The figure shows the least square means, the matrix of adjusted p-values and the diffograms. The diffograms show multiple comparisons of the mean score level categories and indicate the score level (0-3; ABC scores) that significantly differed in the DEMO score. The horizontal and vertical gray reference lines display the means of each score category and, the straight lines represent 95% confidence intervals. Blue lines represent pairs of means that are significantly different from each other at the $p = 0.05$ significance level. Red lines indicate pairs of means that are not significantly different from each other, i.e., those with confidence intervals that intersect the diagonal reference line. These results demonstrate an expected increase in the DEMO score with increased burden of neurological findings.

Supplementary Figure S9

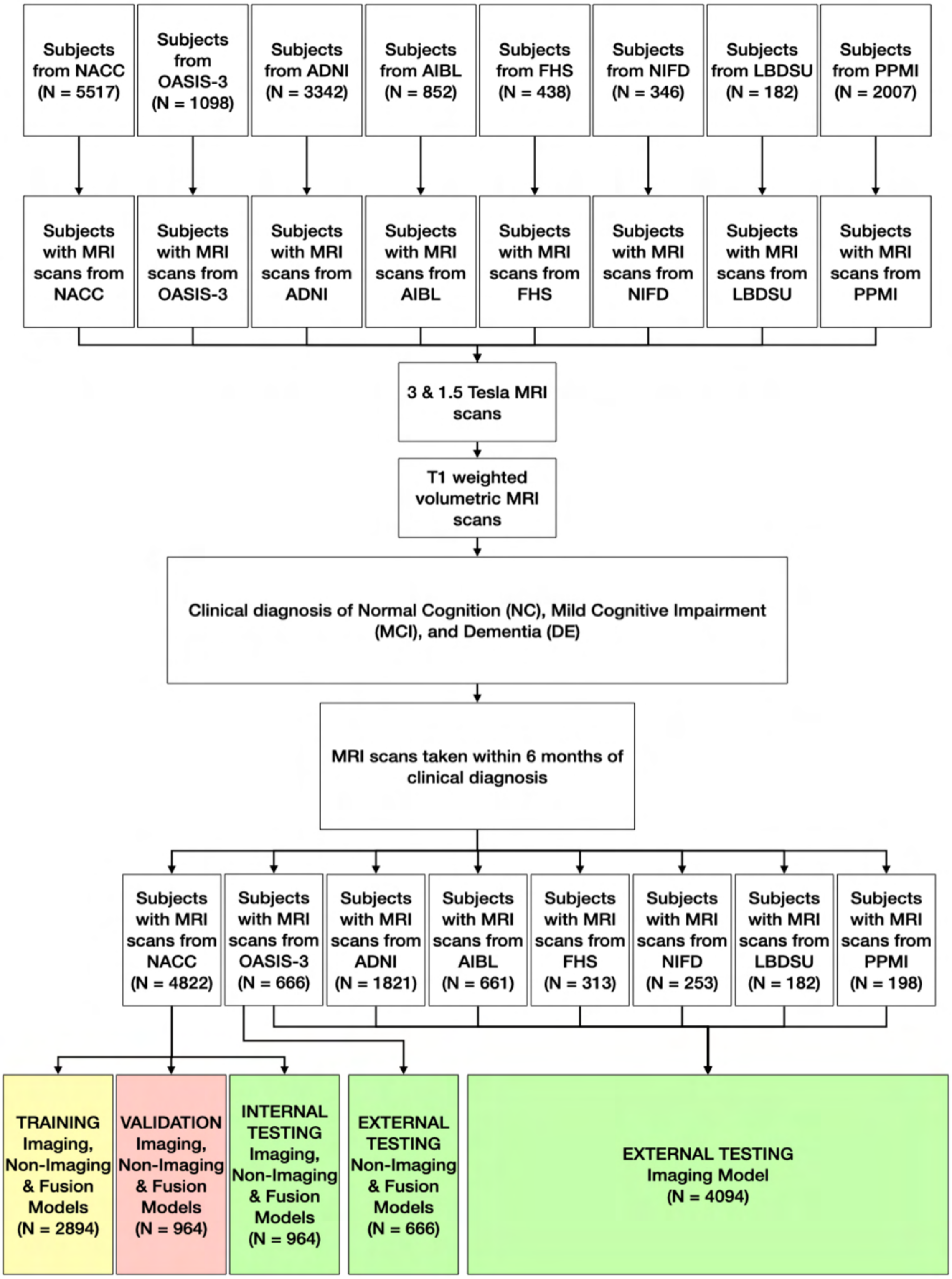

Supplementary Figure S9: Study selection. Data from eight distinct study cohorts contributed towards the model development, validation and testing: The National Alzheimer's Coordinating Center (NACC) dataset (*n*=4,822) , the Open Access Series of Imaging Studies (OASIS-3) dataset (*n*=666), Alzheimer's Disease Neuroimaging Initiative (ADNI) dataset (*n*=1,821), the frontotemporal lobar degeneration neuroimaging initiative (NIFD) dataset (*n*=253), the Parkinson's Progression Marker Initiative (PPMI) dataset (*n*=198), the Australian Imaging, Biomarker and Lifestyle Flagship Study of Ageing (AIBL) dataset (*n*=661), the Framingham Heart Study (FHS) dataset (n=313), and in-house data from the Lewy Body Dementia Center for Excellence at Stanford University (LBDSU) (*n*=182). In each dataset, T1-weighted, 1.5 and 3 Tesla MRIs were selected from participants (See Methods for more details). Only MRIs gathered within 6 months of MCI, AD or non-ADD diagnosis or last confirmed clinical visit (in the case of NC participants) were included for analysis. The NACC data was split in a 3:1:1 ratio for training, validation, and testing the imaging, non-imaging and fusion models; all three the fully trained models were externally tested using the OASIS-3 data and the imaging models were applied to the remaining datasets to assess model generalizability.

Supplementary Figure S10

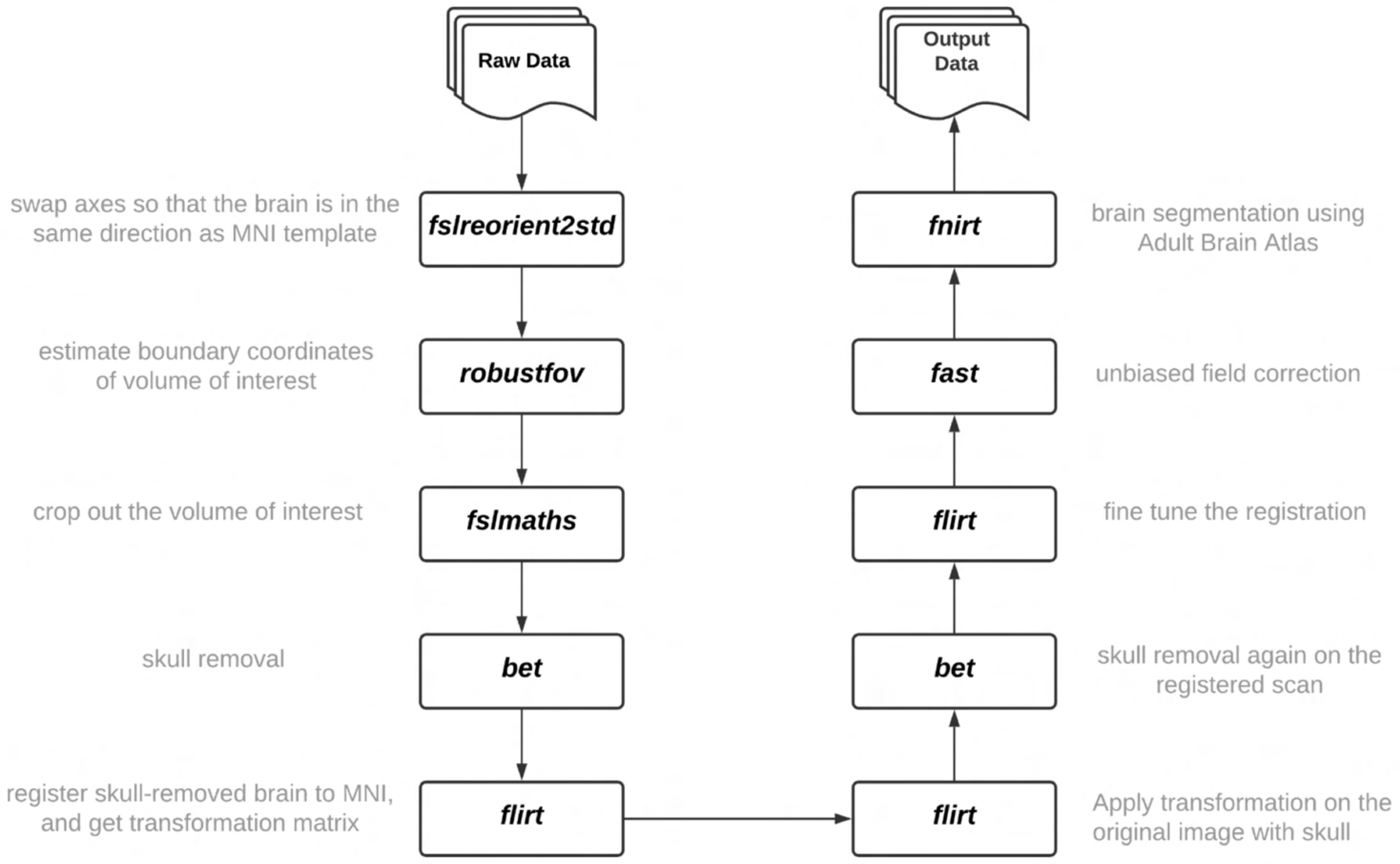

Supplementary Figure S10: MRI preprocessing and segmentation. MRI scans from all datasets were preprocessed using a common pipeline implemented in FSL. Raw MRIs were first reoriented to a standard axis layout and then aligned to the MNI-152 template using a linear registration tool and automatically-identified region-of-interest. These aligned MRIs were then skull-stripped, and the resultant brains then underwent a second linear registration for fine-tuning of MNI alignments, as well as bias field correction for magnetic field inhomogeneities. Finally, specific brain regions were segmented by aligning the Hammersmith Adult brain atlas to registered brains using a non-linear registration. All processed MRIs were inspected visually, and individual brain extraction parameters were adjusted as needed for cases with failed registration. All FSL commands for the above steps are listed within boxes in the accompanying figure.

Supplementary Figure S11

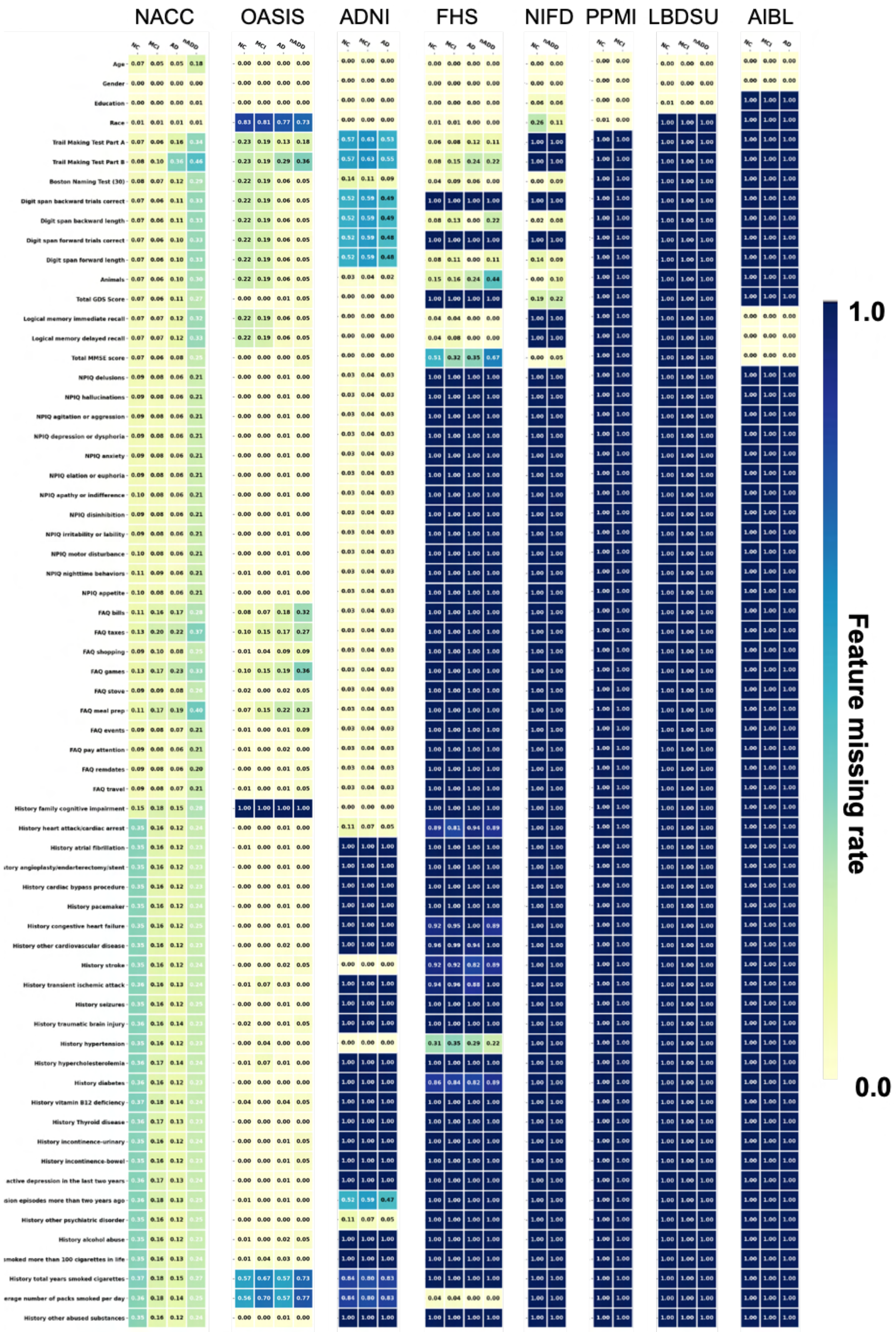

Supplementary Figure S11: Non-imaging feature missing rate. The proportion of missing data is shown for all non-imaging features across the eight cohorts. A value of 0.0 represents that no data is missing, while a value of 1.0 indicates that all data for a particular feature was absent. We further stratify missingness by diagnostic label (NC, MCI, AD, and nADD) in order to demonstrate instances in which data-availability and disease status may be correlated.

Supplementary Figure S12

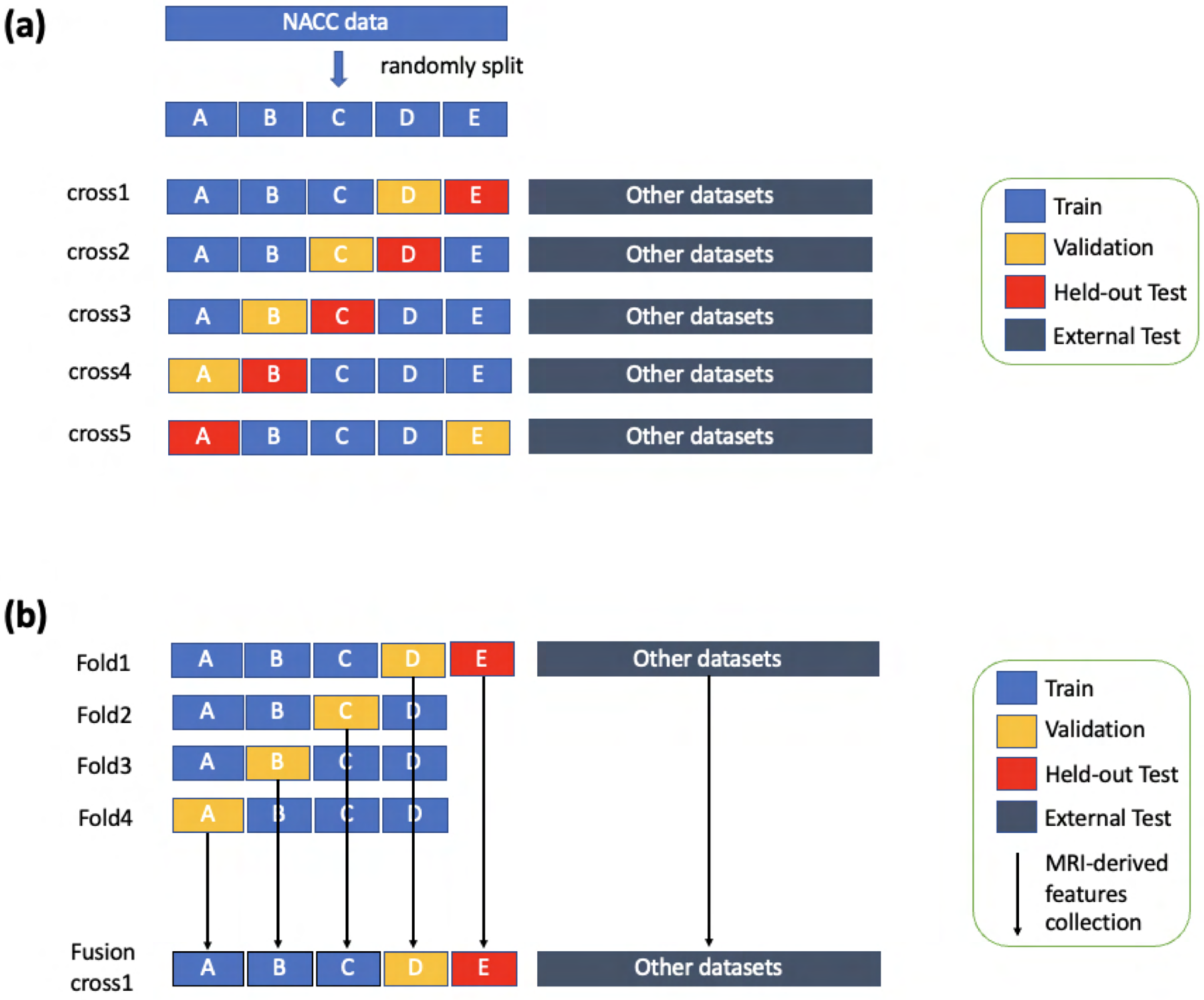

Supplementary Figure S12: Data splitting strategy. (a) A schematic showing the data splitting strategy used for training, validation, and testing of the model is shown. NACC data was split into five folds (labeled A-E) for cross-validation, each of which was enforced to have identical class ratios of NC, MCI, and DE cases. In each cross-validation experiment, different portions of NACC were used for training and validation; all other datasets were kept for external testing. (b) A schematic is shown demonstrating how MRI-derived features were collected for usage in a fusion model. The MRI-derived features used in the fusion model were the direct outcomes from the multi-task CNN model, i.e., raw output from the COG task (i.e., DEMO score) and ALZ score from the AD classification task. To avoid information leakage, we did not collect MRI-derived features from the training part. Instead, we only collected MRI-derived features either from the validation set or the testing set. To collect MRI-derived features from all cases, we shuffled the training and validation parts for four times as illustrated.

Supplementary Table S1

(a)

	COG	COG$_{NC}$	COG$_{MCI}$	COG$_{DE}$	ADD	4-way
Accuracy	0.659±0.016 [0.639-0.679]	0.760±0.009 [0.749-0.771]	0.718±0.025 [0.687-0.749]	0.840±0.011 [0.826-0.854]	0.849±0.012 [0.834-0.864]	0.645±0.015 [0.626-0.664]
F-1	0.569±0.018 [0.547-0.591]	0.797±0.008 [0.787-0.807]	0.308±0.070 [0.221-0.395]	0.603±0.048 [0.543-0.663]	0.914±0.007 [0.905-0.923]	0.466±0.025 [0.435-0.497]
Sensitivity	0.566±0.014 [0.549-0.583]	0.897±0.042 [0.845-0.949]	0.273±0.109 [0.138-0.408]	0.528±0.076 [0.434-0.622]	0.954±0.011 [0.940-0.968]	0.455±0.022 [0.428-0.482]
Specificity	0.802±0.006 [0.795-0.809]	0.610±0.055 [0.542-0.678]	0.861±0.066 [0.779-0.943]	0.935±0.018 [0.913-0.957]	0.286±0.031 [0.248-0.324]	0.848±0.005 [0.842-0.854]
MCC	0.404±0.022 [0.377-0.431]	0.536±0.016 [0.516-0.556]	0.156±0.033 [0.115-0.197]	0.519±0.043 [0.466-0.572]	0.315±0.047 [0.257-0.373]	0.352±0.036 [0.307-0.397]

(b)

	COG	COG$_{NC}$	COG$_{MCI}$	COG$_{DE}$	ADD	4-way
Accuracy	0.690±0.039 [0.642-0.738]	0.785±0.014 [0.768-0.802]	0.798±0.056 [0.728-0.868]	0.796±0.011 [0.782-0.810]	0.833±0.030 [0.796-0.870]	0.674±0.034 [0.632-0.716]
F-1	0.503±0.014 [0.486-0.520]	0.831±0.015 [0.812-0.850]	0.084±0.019 [0.060-0.108]	0.592±0.046 [0.535-0.649]	0.905±0.020 [0.880-0.930]	0.396±0.021 [0.370-0.422]
Sensitivity	0.514±0.010 [0.502-0.526]	0.833±0.038 [0.786-0.880]	0.244±0.121 [0.094-0.394]	0.464±0.063 [0.386-0.542]	0.893±0.043 [0.840-0.946]	0.401±0.006 [0.394-0.408]
Specificity	0.826±0.006 [0.819-0.833]	0.702±0.035 [0.659-0.745]	0.822±0.064 [0.743-0.901]	0.954±0.015 [0.935-0.973]	0.309±0.088 [0.200-0.418]	0.866±0.004 [0.861-0.871]
MCC	0.359±0.006 [0.352-0.366]	0.537±0.021 [0.511-0.563]	0.032±0.026 [-0.000-0.064]	0.510±0.028 [0.475-0.545]	0.188±0.027 [0.154-0.222]	0.287±0.016 [0.267-0.307]

(c)

	COG	COG$_{NC}$	COG$_{MCI}$	COG$_{DE}$	ADD	4-way
Accuracy	0.593±0.008 [0.583-0.603]	0.695±0.005 [0.689-0.701]	0.657±0.019 [0.633-0.681]	0.832±0.004 [0.827-0.837]	0.837±0.012 [0.822-0.852]	0.583±0.007 [0.574-0.592]
F-1	0.516±0.010 [0.504-0.528]	0.736±0.008 [0.726-0.746]	0.279±0.069 [0.193-0.365]	0.534±0.036 [0.489-0.579]	0.900±0.008 [0.890-0.910]	0.450±0.022 [0.423-0.477]
Sensitivity	0.523±0.007 [0.514-0.532]	0.860±0.043 [0.807-0.913]	0.232±0.091 [0.119-0.345]	0.478±0.068 [0.394-0.562]	0.929±0.026 [0.897-0.961]	0.445±0.027 [0.411-0.479]
Specificity	0.766±0.001 [0.765-0.767]	0.534±0.050 [0.472-0.596]	0.842±0.066 [0.760-0.924]	0.923±0.019 [0.899-0.947]	0.489±0.101 [0.364-0.614]	0.823±0.001 [0.822-0.824]
MCC	0.316±0.007 [0.307-0.325]	0.419±0.008 [0.409-0.429]	0.088±0.018 [0.066-0.110]	0.442±0.024 [0.412-0.472]	0.467±0.053 [0.401-0.533]	0.306±0.028 [0.271-0.341]

Supplementary Table S1: MRI-only model performance. The performance metrics, including accuracy, F-1 score, sensitivity, specificity and MCC, were evaluated on various tasks where MRI scans were the sole inputs to the deep learning model. Results for the (a) NACC test set, (b) OASIS and (c) combined external datasets are shown. For each table, the columns (COG$_{NC}$, COG$_{MCI}$, COG$_{DE}$) correspond to metrics for a one-versus-rest classification task in which the goal was to individually delineate these three cognitive categories from all others within the overarching COG task. The "COG" column corresponds to the complete COG task of separating each NC/MCI/DE category (i.e., a 3-way classification). The "ADD" columns corresponds to the task of classifying AD and nADD diagnosis given that a DE diagnosis has already been obtained from the COG task. Lastly, the "4-way" column corresponds to the complete classification workflow in which NC, MCI, AD, and nADD cases are delineated in a final four-way classification.

Supplementary Table S2

(a)

	COG	COG$_{NC}$	COG$_{MCI}$	COG$_{DE}$	ADD	4-way
**Accuracy**	0.782±0.011 [0.769-0.796]	0.856±0.008 [0.846-0.866]	0.790±0.010 [0.777-0.803]	0.919±0.006 [0.912-0.926]	0.806±0.033 [0.765-0.847]	0.748±0.012 [0.734-0.763]
**F-1**	0.752±0.013 [0.736-0.768]	0.862±0.009 [0.852-0.873]	0.566±0.022 [0.539-0.593]	0.827±0.012 [0.812-0.842]	0.884±0.019 [0.860-0.908]	0.611±0.027 [0.577-0.645]
**Sensitivity**	0.752±0.013 [0.736-0.768]	0.863±0.016 [0.843-0.883]	0.563±0.032 [0.524-0.603]	0.831±0.020 [0.806-0.856]	0.878±0.019 [0.854-0.901]	0.612±0.029 [0.575-0.648]
**Specificity**	0.886±0.006 [0.879-0.893]	0.848±0.014 [0.831-0.865]	0.863±0.013 [0.846-0.880]	0.946±0.007 [0.937-0.955]	0.417±0.140 [0.244-0.590]	0.906±0.004 [0.900-0.911]
**MCC**	0.638±0.018 [0.616-0.660]	0.711±0.016 [0.691-0.732]	0.428±0.027 [0.395-0.462]	0.775±0.016 [0.755-0.794]	0.283±0.139 [0.110-0.456]	0.517±0.031 [0.478-0.555]

(b)

	COG	COG$_{NC}$	COG$_{MCI}$	COG$_{DE}$	ADD	4-way
**Accuracy**	0.769±0.019 [0.745-0.793]	0.862±0.013 [0.845-0.878]	0.780±0.022 [0.753-0.808]	0.895±0.005 [0.889-0.901]	0.786±0.014 [0.769-0.804]	0.720±0.019 [0.696-0.745]
**F-1**	0.613±0.008 [0.603-0.622]	0.884±0.013 [0.867-0.900]	0.143±0.006 [0.136-0.150]	0.811±0.011 [0.797-0.825]	0.875±0.010 [0.863-0.886]	0.480±0.011 [0.466-0.494]
**Sensitivity**	0.658±0.008 [0.648-0.668]	0.825±0.026 [0.794-0.857]	0.452±0.049 [0.391-0.513]	0.697±0.019 [0.673-0.721]	0.832±0.019 [0.809-0.855]	0.518±0.006 [0.511-0.525]
**Specificity**	0.903±0.005 [0.897-0.909]	0.926±0.009 [0.915-0.936]	0.794±0.025 [0.764-0.825]	0.990±0.004 [0.985-0.995]	0.382±0.068 [0.297-0.466]	0.914±0.004 [0.909-0.918]
**MCC**	0.535±0.009 [0.524-0.546]	0.727±0.020 [0.703-0.752]	0.118±0.010 [0.105-0.131]	0.761±0.011 [0.748-0.774]	0.165±0.043 [0.112-0.218]	0.411±0.011 [0.398-0.424]

Supplementary Table S2: Non-imaging model performance. After compiling and comparing the performance of numerous machine learning classifiers on non-imaging data, we determined that the CatBoost algorithm yielded the strongest overall performance across all classification tasks. We highlight the performance metrics of this model when trained on non-imaging data only and tested on (a) NACC test set and (b) OASIS. Here, F-1 score, sensitivity, specificity, and MCC are reported for various prediction tasks. For each table, the columns (COG$_{NC}$, COG$_{MCI}$, COG$_{DE}$) correspond to metrics for a one-versus-rest classification task in which the goal was to individually delineate these three cognitive categories from all others within the overarching COG task. The "COG" column corresponds to the complete COG task of separating each NC/MCI/DE category (i.e., a 3-way classification). The "ADD" column corresponds to the task of detecting AD diagnosis given that a DE diagnosis has already been obtained from the COG task. Lastly, the "4-way" column corresponds to the complete classification workflow in which NC, MCI, AD, and nADD cases are delineated in a final four-way classification.

Supplementary Table S3

Supplementary Table S3: Fusion model performance on the NACC dataset. The performance of multiple types of fusion models was assessed on the NACC test set across all prediction tasks. In addition to the combination of CNN-derived features and a CatBoost model, we also combined our CNN with (a) decision tree (b) K-nearest neighbor (c) multilayer perceptron (d) random forest (e) support vector machine and (f) XGBoost model. The F-1 score, sensitivity, specificity, and MCC values are reported for various predictions. The column names have identical meaning to those described in prior tables. As previously mentioned, we judged that these models offered lesser performance than a CatBoost fusion model, and thus these additional classifiers were not used in the final fusion.

(a)

	COG	COG$_{NC}$	COG$_{MCI}$	COG$_{DE}$	ADD	4-way
**Accuracy**	0.698±0.030 [0.661-0.735]	0.797±0.023 [0.768-0.826]	0.722±0.022 [0.695-0.749]	0.878±0.017 [0.857-0.899]	0.731±0.072 [0.642-0.820]	0.654±0.032 [0.614-0.694]
**F-1**	0.665±0.027 [0.631-0.699]	0.801±0.025 [0.770-0.832]	0.461±0.016 [0.441-0.481]	0.734±0.045 [0.678-0.790]	0.832±0.051 [0.769-0.895]	0.513±0.028 [0.478-0.548]
**Sensitivity**	0.666±0.028 [0.631-0.701]	0.783±0.037 [0.737-0.829]	0.487±0.023 [0.458-0.516]	0.729±0.066 [0.647-0.811]	0.803±0.092 [0.689-0.917]	0.516±0.031 [0.478-0.554]
**Specificity**	0.844±0.014 [0.827-0.861]	0.813±0.014 [0.796-0.830]	0.798±0.034 [0.756-0.840]	0.923±0.008 [0.913-0.933]	0.343±0.105 [0.213-0.473]	0.872±0.011 [0.858-0.886]
**MCC**	0.509±0.040 [0.459-0.559]	0.596±0.044 [0.541-0.651]	0.276±0.029 [0.240-0.312]	0.655±0.054 [0.588-0.722]	0.149±0.136 [-0.020-0.318]	0.388±0.040 [0.338-0.438]

(b)

	COG	COG$_{NC}$	COG$_{MCI}$	COG$_{DE}$	ADD	4-way
**Accuracy**	0.742±0.006 [0.735-0.749]	0.812±0.008 [0.802-0.822]	0.763±0.005 [0.757-0.769]	0.909±0.007 [0.900-0.918]	0.838±0.006 [0.831-0.845]	0.714±0.006 [0.707-0.721]
**F-1**	0.666±0.011 [0.652-0.680]	0.838±0.009 [0.827-0.849]	0.354±0.038 [0.307-0.401]	0.807±0.014 [0.790-0.824]	0.911±0.004 [0.906-0.916]	0.493±0.009 [0.482-0.504]
**Sensitivity**	0.671±0.007 [0.662-0.680]	0.931±0.029 [0.895-0.967]	0.269±0.046 [0.212-0.326]	0.814±0.016 [0.794-0.834]	0.983±0.015 [0.964-1.002]	0.509±0.006 [0.502-0.516]
**Specificity**	0.847±0.003 [0.843-0.851]	0.681±0.028 [0.646-0.716]	0.923±0.021 [0.897-0.949]	0.938±0.008 [0.928-0.948]	0.051±0.049 [-0.010-0.112]	0.878±0.003 [0.874-0.882]
**MCC**	0.545±0.008 [0.535-0.555]	0.637±0.019 [0.613-0.661]	0.251±0.014 [0.234-0.268]	0.748±0.018 [0.726-0.770]	0.073±0.057 [0.002-0.144]	0.405±0.006 [0.398-0.412]

(c)

	COG	COG$_{NC}$	COG$_{MCI}$	COG$_{DE}$	ADD	4-way
**Accuracy**	0.730±0.018 [0.708-0.752]	0.822±0.015 [0.803-0.841]	0.754±0.019 [0.730-0.778]	0.883±0.010 [0.871-0.895]	0.853±0.021 [0.827-0.879]	0.706±0.015 [0.687-0.725]
**F-1**	0.658±0.024 [0.628-0.688]	0.841±0.011 [0.827-0.855]	0.373±0.048 [0.313-0.433]	0.760±0.021 [0.734-0.786]	0.918±0.011 [0.904-0.932]	0.521±0.030 [0.484-0.558]
**Sensitivity**	0.666±0.028 [0.631-0.701]	0.899±0.012 [0.884-0.914]	0.301±0.044 [0.246-0.356]	0.797±0.068 [0.713-0.881]	0.977±0.005 [0.971-0.983]	0.528±0.027 [0.494-0.562]
**Specificity**	0.849±0.013 [0.833-0.865]	0.738±0.039 [0.690-0.786]	0.900±0.023 [0.871-0.929]	0.909±0.024 [0.879-0.939]	0.183±0.123 [0.030-0.336]	0.881±0.009 [0.870-0.892]
**MCC**	0.526±0.036 [0.481-0.571]	0.649±0.028 [0.614-0.684]	0.243±0.057 [0.172-0.314]	0.687±0.029 [0.651-0.723]	0.251±0.157 [0.056-0.446]	0.425±0.032 [0.385-0.465]

(d)

	COG	COG$_{NC}$	COG$_{MCI}$	COG$_{DE}$	ADD	4-way
**Accuracy**	0.794±0.009 [0.783-0.805]	0.866±0.010 [0.854-0.878]	0.799±0.008 [0.789-0.809]	0.922±0.007 [0.913-0.931]	0.848±0.015 [0.829-0.867]	0.770±0.008 [0.760-0.780]
**F-1**	0.755±0.014 [0.738-0.772]	0.877±0.007 [0.868-0.886]	0.562±0.038 [0.515-0.609]	0.826±0.022 [0.799-0.853]	0.914±0.008 [0.904-0.924]	0.597±0.030 [0.560-0.634]
**Sensitivity**	0.748±0.015 [0.729-0.767]	0.913±0.023 [0.884-0.942]	0.535±0.079 [0.437-0.633]	0.796±0.055 [0.728-0.864]	0.964±0.018 [0.942-0.986]	0.590±0.021 [0.564-0.616]
**Specificity**	0.886±0.007 [0.877-0.895]	0.814±0.039 [0.766-0.862]	0.884±0.029 [0.848-0.920]	0.961±0.010 [0.949-0.973]	0.217±0.103 [0.089-0.345]	0.908±0.005 [0.902-0.914]
**MCC**	0.650±0.016 [0.630-0.670]	0.734±0.017 [0.713-0.755]	0.438±0.030 [0.401-0.475]	0.779±0.023 [0.750-0.808]	0.261±0.106 [0.129-0.393]	0.522±0.035 [0.479-0.565]

(e)

	COG	COG$_{NC}$	COG$_{MCI}$	COG$_{DE}$	ADD	4-way
**Accuracy**	0.784±0.010 [0.772-0.796]	0.856±0.013 [0.840-0.872]	0.792±0.009 [0.781-0.803]	0.920±0.007 [0.911-0.929]	0.858±0.008 [0.848-0.868]	0.760±0.007 [0.751-0.769]
**F-1**	0.742±0.016 [0.722-0.762]	0.868±0.010 [0.856-0.880]	0.529±0.053 [0.463-0.595]	0.828±0.020 [0.803-0.853]	0.922±0.004 [0.917-0.927]	0.577±0.018 [0.555-0.599]
**Sensitivity**	0.740±0.016 [0.720-0.760]	0.902±0.026 [0.870-0.934]	0.490±0.096 [0.371-0.609]	0.826±0.055 [0.758-0.894]	0.990±0.006 [0.983-0.997]	0.577±0.014 [0.560-0.594]
**Specificity**	0.881±0.008 [0.871-0.891]	0.805±0.043 [0.752-0.858]	0.889±0.031 [0.851-0.927]	0.949±0.008 [0.939-0.959]	0.137±0.055 [0.069-0.205]	0.904±0.006 [0.897-0.911]
**MCC**	0.632±0.019 [0.608-0.656]	0.714±0.025 [0.683-0.745]	0.406±0.043 [0.353-0.459]	0.777±0.026 [0.745-0.809]	0.269±0.082 [0.167-0.371]	0.510±0.023 [0.481-0.539]

(f)

	COG	COG$_{NC}$	COG$_{MCI}$	COG$_{DE}$	ADD	4-way
**Accuracy**	0.784±0.013 [0.768-0.800]	0.857±0.007 [0.848-0.866]	0.792±0.010 [0.780-0.804]	0.920±0.010 [0.908-0.932]	0.842±0.009 [0.831-0.853]	0.759±0.012 [0.744-0.774]
**F-1**	0.748±0.018 [0.726-0.770]	0.868±0.006 [0.861-0.875]	0.555±0.026 [0.523-0.587]	0.821±0.027 [0.787-0.855]	0.910±0.006 [0.903-0.917]	0.596±0.037 [0.550-0.642]
**Sensitivity**	0.742±0.023 [0.713-0.771]	0.894±0.018 [0.872-0.916]	0.534±0.031 [0.496-0.572]	0.798±0.053 [0.732-0.864]	0.949±0.029 [0.913-0.985]	0.592±0.036 [0.547-0.637]
**Specificity**	0.882±0.009 [0.871-0.893]	0.816±0.027 [0.782-0.850]	0.874±0.008 [0.864-0.884]	0.956±0.006 [0.949-0.963]	0.263±0.161 [0.063-0.463]	0.905±0.006 [0.898-0.912]
**MCC**	0.635±0.024 [0.605-0.665]	0.715±0.013 [0.699-0.731]	0.420±0.031 [0.382-0.458]	0.771±0.031 [0.733-0.809]	0.260±0.121 [0.110-0.410]	0.512±0.036 [0.467-0.557]

Supplementary Table S4

(a)

	COG	COG_{NC}	COG_{MCI}	COG_{DE}	ADD	4-way
**Accuracy**	0.670±0.034<br>[0.628-0.712]	0.775±0.035<br>[0.731-0.819]	0.722±0.032<br>[0.681-0.762]	0.844±0.013<br>[0.828-0.859]	0.752±0.071<br>[0.663-0.840]	0.622±0.034<br>[0.580-0.664]
**F-1**	0.542±0.014<br>[0.525-0.559]	0.801±0.038<br>[0.753-0.848]	0.111±0.035<br>[0.068-0.155]	0.714±0.029<br>[0.678-0.751]	0.850±0.052<br>[0.785-0.914]	0.400±0.018<br>[0.378-0.423]
**Sensitivity**	0.587±0.040<br>[0.538-0.637]	0.716±0.060<br>[0.642-0.790]	0.437±0.151<br>[0.250-0.624]	0.608±0.047<br>[0.550-0.667]	0.799±0.089<br>[0.688-0.910]	0.437±0.040<br>[0.387-0.486]
**Specificity**	0.856±0.011<br>[0.842-0.869]	0.878±0.018<br>[0.856-0.900]	0.734±0.036<br>[0.689-0.778]	0.956±0.020<br>[0.931-0.980]	0.336±0.121<br>[0.187-0.486]	0.880±0.009<br>[0.868-0.891]
**MCC**	0.427±0.023<br>[0.398-0.455]	0.573±0.051<br>[0.510-0.637]	0.075±0.061<br>[-0.002-0.151]	0.632±0.031<br>[0.593-0.671]	0.103±0.058<br>[0.031-0.174]	0.307±0.027<br>[0.273-0.340]

(b)

	COG	COG_{NC}	COG_{MCI}	COG_{DE}	ADD	4-way
**Accuracy**	0.818±0.017<br>[0.797-0.839]	0.881±0.011<br>[0.868-0.895]	0.864±0.025<br>[0.832-0.895]	0.891±0.005<br>[0.884-0.897]	0.876±0.023<br>[0.847-0.905]	0.797±0.016<br>[0.777-0.816]
**F-1**	0.602±0.009<br>[0.592-0.613]	0.908±0.010<br>[0.895-0.921]	0.097±0.033<br>[0.055-0.138]	0.803±0.010<br>[0.790-0.815]	0.934±0.013<br>[0.917-0.950]	0.445±0.006<br>[0.438-0.452]
**Sensitivity**	0.599±0.022<br>[0.572-0.626]	0.924±0.033<br>[0.882-0.965]	0.185±0.088<br>[0.076-0.294]	0.688±0.014<br>[0.671-0.706]	0.974±0.026<br>[0.942-1.006]	0.451±0.016<br>[0.430-0.471]
**Specificity**	0.895±0.003<br>[0.892-0.899]	0.807±0.036<br>[0.762-0.851]	0.892±0.029<br>[0.857-0.928]	0.987±0.006<br>[0.980-0.994]	0.018±0.022<br>[-0.009-0.046]	0.914±0.002<br>[0.912-0.917]
**MCC**	0.513±0.010<br>[0.501-0.526]	0.743±0.021<br>[0.716-0.769]	0.048±0.042<br>[-0.004-0.099]	0.750±0.013<br>[0.733-0.766]	-0.008±0.047<br>[-0.066-0.051]	0.373±0.005<br>[0.366-0.380]

(c)

	COG	COG_{NC}	COG_{MCI}	COG_{DE}	ADD	4-way
**Accuracy**	0.762±0.028<br>[0.727-0.796]	0.841±0.013<br>[0.825-0.858]	0.805±0.031<br>[0.767-0.843]	0.877±0.024<br>[0.847-0.906]	0.874±0.020<br>[0.849-0.899]	0.739±0.026<br>[0.707-0.771]
**F-1**	0.595±0.026<br>[0.563-0.627]	0.870±0.011<br>[0.856-0.884]	0.142±0.017<br>[0.121-0.164]	0.773±0.058<br>[0.701-0.845]	0.932±0.012<br>[0.917-0.946]	0.467±0.027<br>[0.433-0.501]
**Sensitivity**	0.630±0.030<br>[0.593-0.668]	0.834±0.017<br>[0.813-0.855]	0.393±0.038<br>[0.346-0.439]	0.664±0.090<br>[0.553-0.776]	0.956±0.022<br>[0.929-0.984]	0.489±0.026<br>[0.456-0.521]
**Specificity**	0.885±0.012<br>[0.870-0.899]	0.854±0.019<br>[0.830-0.878]	0.823±0.033<br>[0.782-0.863]	0.978±0.013<br>[0.962-0.994]	0.155±0.036<br>[0.109-0.200]	0.907±0.008<br>[0.896-0.917]
**MCC**	0.499±0.029<br>[0.463-0.535]	0.672±0.026<br>[0.640-0.704]	0.111±0.023<br>[0.082-0.139]	0.715±0.055<br>[0.647-0.783]	0.161±0.070<br>[0.074-0.248]	0.398±0.033<br>[0.356-0.439]

(d)

	COG	COG_{NC}	COG_{MCI}	COG_{DE}	ADD	4-way
**Accuracy**	0.728±0.047<br>[0.669-0.786]	0.848±0.042<br>[0.796-0.900]	0.741±0.053<br>[0.675-0.807]	0.867±0.013<br>[0.850-0.883]	0.884±0.011<br>[0.871-0.897]	0.709±0.048<br>[0.649-0.768]
**F-1**	0.582±0.020<br>[0.557-0.607]	0.869±0.043<br>[0.816-0.922]	0.133±0.016<br>[0.113-0.152]	0.743±0.033<br>[0.702-0.784]	0.938±0.006<br>[0.930-0.945]	0.445±0.027<br>[0.411-0.479]
**Sensitivity**	0.630±0.018<br>[0.608-0.652]	0.808±0.079<br>[0.710-0.906]	0.481±0.062<br>[0.405-0.558]	0.600±0.041<br>[0.549-0.651]	0.977±0.015<br>[0.959-0.996]	0.481±0.018<br>[0.458-0.504]
**Specificity**	0.888±0.012<br>[0.873-0.902]	0.918±0.024<br>[0.888-0.948]	0.751±0.057<br>[0.680-0.823]	0.994±0.001<br>[0.993-0.995]	0.064±0.036<br>[0.018-0.109]	0.910±0.009<br>[0.898-0.921]
**MCC**	0.503±0.024<br>[0.473-0.534]	0.707±0.060<br>[0.633-0.781]	0.107±0.021<br>[0.081-0.132]	0.697±0.031<br>[0.658-0.735]	0.071±0.049<br>[0.010-0.132]	0.387±0.036<br>[0.342-0.431]

(e)

	COG	COG_{NC}	COG_{MCI}	COG_{DE}	ADD	4-way
**Accuracy**	0.791±0.037<br>[0.745-0.837]	0.879±0.022<br>[0.851-0.907]	0.808±0.042<br>[0.755-0.860]	0.896±0.014<br>[0.879-0.913]	0.889±0.006<br>[0.882-0.897]	0.773±0.035<br>[0.729-0.816]
**F-1**	0.632±0.019<br>[0.608-0.655]	0.899±0.021<br>[0.873-0.926]	0.185±0.014<br>[0.168-0.202]	0.811±0.031<br>[0.772-0.850]	0.941±0.003<br>[0.937-0.945]	0.473±0.008<br>[0.463-0.483]
**Sensitivity**	0.695±0.019<br>[0.671-0.719]	0.856±0.046<br>[0.799-0.913]	0.533±0.106<br>[0.401-0.665]	0.696±0.051<br>[0.632-0.759]	0.988±0.007<br>[0.979-0.996]	0.527±0.013<br>[0.511-0.543]
**Specificity**	0.910±0.009<br>[0.899-0.921]	0.919±0.020<br>[0.895-0.943]	0.819±0.048<br>[0.760-0.879]	0.991±0.005<br>[0.985-0.997]	0.027±0.036 [-<br>0.018-0.072]	0.926±0.006<br>[0.919-0.933]
**MCC**	0.566±0.020<br>[0.541-0.590]	0.756±0.035<br>[0.713-0.800]	0.177±0.021<br>[0.151-0.204]	0.763±0.031<br>[0.725-0.801]	0.026±0.082 [-<br>0.076-0.127]	0.423±0.008<br>[0.413-0.432]

(f)

	COG	COG_{NC}	COG_{MCI}	COG_{DE}	ADD	4-way
**Accuracy**	0.719±0.034<br>[0.677-0.761]	0.824±0.044<br>[0.770-0.878]	0.739±0.036<br>[0.694-0.785]	0.875±0.015<br>[0.856-0.894]	0.856±0.038<br>[0.809-0.903]	0.697±0.039<br>[0.649-0.745]
**F-1**	0.582±0.007<br>[0.574-0.591]	0.847±0.045<br>[0.790-0.903]	0.136±0.013<br>[0.120-0.151]	0.765±0.036<br>[0.720-0.809]	0.919±0.024<br>[0.889-0.949]	0.460±0.012<br>[0.445-0.476]
**Sensitivity**	0.638±0.015<br>[0.619-0.657]	0.777±0.079<br>[0.679-0.875]	0.504±0.080<br>[0.405-0.603]	0.633±0.051<br>[0.570-0.695]	0.924±0.060<br>[0.849-0.999]	0.499±0.016<br>[0.480-0.519]
**Specificity**	0.882±0.009<br>[0.871-0.893]	0.907±0.022<br>[0.879-0.934]	0.749±0.041<br>[0.699-0.800]	0.991±0.004<br>[0.986-0.995]	0.255±0.179<br>[0.033-0.476]	0.905±0.008<br>[0.895-0.915]
**MCC**	0.497±0.013<br>[0.481-0.513]	0.663±0.064<br>[0.583-0.743]	0.114±0.022<br>[0.086-0.142]	0.715±0.034<br>[0.672-0.757]	0.181±0.101<br>[0.055-0.307]	0.398±0.022<br>[0.371-0.424]

Supplementary Table S4: Fusion model performance on the OASIS dataset. The performance of multiple types of fusion models was assessed on the OASIS dataset across all prediction tasks. In addition to the combination of CNN-derived features and a CatBoost model, we also combined our CNN with (a) decision tree (b) K-nearest neighbor (c) multilayer perceptron (d) random forest (e) support vector machine and (f) XGBoost model. The F-1 score, sensitivity, specificity, and MCC values are reported for various predictions. The column names have identical meaning to those described in prior tables.

Supplementary Table S5

index	Adult brain atlas [region]	Sagittal view [node index]	axial view [node index]
1	TL hippocampus R	21	37
2	TL hippocampus L		38
3	TL amygdala R	18	29
4	TL amygdala L		30
5	TL anterior temporal lobe medial part R	19	33
6	TL anterior temporal lobe medial part L		34
7	TL anterior temporal lobe lateral part R		31
8	TL anterior temporal lobe lateral part L		32
9	TL parahippocampal and ambient gyrus R	23	41
10	TL parahippocampal and ambient gyrus L		42
11	TL superior temporal gyrus middle part R		47
12	TL superior temporal gyrus middle part L		48
13	TL middle and inferior temporal gyrus R	22	39
14	TL middle and inferior temporal gyrus L		40
15	TL fusiform gyrus R	20	35
16	TL fusiform gyrus L		36
17	cerebellum R	27	
18	cerebellum L		
19	brainstem excluding substantia nigra	25	
20	insula posterior long gyrus L	29	
21	insula posterior long gyrus R		
22	OL lateral remainder occipital lobe L	12	26
23	OL lateral remainder occipital lobe R		25
24	CG anterior cingulate gyrus L	1	
25	CG anterior cingulate gyrus R		
26	CG posterior cingulate gyrus L	2	2
27	CG posterior cingulate gyrus R		1
28	FL middle frontal gyrus L	4	
29	FL middle frontal gyrus R		
30	TL posterior temporal lobe L		44
31	TL posterior temporal lobe R		43

Supplementary Table S5: Definition of network nodes. In **Figs. 4d & 4e**, we presented the network visualization of the inter-region correlation structure of the brain from the axial and sagittal views. The node was defined to represent a particular region from the parcellated brain and the edges between nodes demonstrated the sign and degree of correlation between a pair of nodes. We parcellated the brain MRI into 95 regions using the segmentation mask provided from the Hammersmith Adult Brain Atlas. To project the 3D structure into a axial and sagittal plane, we redefined the node for best visualization purpose. The “index” and “Adult brain atlas” columns show the completed 95 structures and their corresponding indexes from the Hammersmith Adult Brain Atlas. The “sagittal view” and “axial view” columns demonstrate how we merged and re-indexed regions and the node index number is what we labeled each node from **Figs. 4d & 4e**, respectively. In the sagittal view, we focused on visualizing the correlation between the temporal lobe, frontal lobe, parietal lobe, occipital lobe, cerebellum and brainstem. Specifically, we merged the same structures from the left and right hemisphere as a single node in the sagittal projection, thus ending up with a total of 33 final nodes as defined in this table. In the axial view, we excluded some of the structures that have been already shown in the sagittal view, for example, insula, the third ventricle, etc. The focus of the axial view is to reveal the correlation between cerebrum structures from the left and right hemispheres. Our selection of the axial nodes yielded 57 regions.

Supplementary Table S5 continued...

index	Adult brain atlas [region]	Sagittal view [node index]	axial view [node index]
32	PL angular gyrus L	14	
33	PL angular gyrus R		
34	caudate nucleus L	26	50
35	caudate nucleus R		49
36	nucleus accumbens L	30	
37	nucleus accumbens R		
38	putamen L	31	55
39	putamen R		54
40	thalamus L	33	57
41	thalamus R		56
42	pallidum L		53
43	pallidum R		52
44	corpus callosum	28	51
45	Lateral ventricle excluding temporal horn R	9	
46	Lateral ventricle excluding temporal horn L		
47	Lateral ventricle temporal horn R	10	
48	Lateral ventricle temporal horn L		
49	Third ventricle	24	
50	FL precentral gyrus L	6	16
51	FL precentral gyrus R		15
52	FL straight gyrus L	7	18
53	FL straight gyrus R		17
54	FL anterior orbital gyrus L	3	4
55	FL anterior orbital gyrus R		3
56	FL inferior frontal gyrus L		6
57	FL inferior frontal gyrus R		5
58	FL superior frontal gyrus L	8	22
59	FL superior frontal gyrus R		21
60	PL postcentral gyrus L	15	
61	PL postcentral gyrus R		
62	PL superior parietal gyrus L	16	
63	PL superior parietal gyrus R		
64	OL lingual gyrus L	13	28
65	OL lingual gyrus R		27
66	OL cuneus L	11	24
67	OL cuneus R		23
68	FL medial orbital gyrus L	3	10
69	FL medial orbital gyrus R		9
70	FL lateral orbital gyrus L		8
71	FL lateral orbital gyrus R		7
72	FL posterior orbital gyrus L		12
73	FL posterior orbital gyrus R		11
74	substantia nigra L	32	
75	substantia nigra L		
76	FL subgenual frontal cortex L		20
77	FL subgenual frontal cortex R		19
78	FL subcallosal area L		
79	FL subcallosal area R		
80	FL pre-subgenual frontal cortex L	5	14
81	FL pre-subgenual frontal cortex R		13
82	TL superior temporal gyrus anterior part L		46
83	TL superior temporal gyrus anterior part R		45
84	PL supramarginal gyrus L	17	
85	PL supramarginal gyrus R		
86	insula anterior short gyrus L	29	
87	insula anterior short gyrus R		
88	insula middle short gyrus L		
89	insula middle short gyrus R		
90	insula posterior short gyrus L		
91	insula posterior short gyrus R		
92	insula anterior inferior cortex L		
93	insula anterior inferior cortex R		
94	insula anterior long gyrus L		
95	insula anterior long gyrus R		

Supplementary Table S6

(a)

	COG	COG$_{NC}$	COG$_{MCI}$	COG$_{DE}$	ADD	4-way
Accuracy	0.713±0.093 [0.665-0.761]	0.849±0.067 [0.815-0.883]	0.744±0.067 [0.710-0.778]	0.834±0.070 [0.798-0.870]	0.624±0.080 [0.583-0.665]	0.565±0.070 [0.529-0.601]
F-1	0.678±0.089 [0.632-0.724]	0.739±0.064 [0.706-0.772]	0.486±0.123 [0.423-0.549]	0.811±0.107 [0.756-0.866]	0.605±0.100 [0.554-0.656]	0.549±0.074 [0.511-0.587]
Sensitivity	0.695±0.072 [0.658-0.732]	0.821±0.113 [0.763-0.879]	0.494±0.163 [0.410-0.578]	0.768±0.170 [0.681-0.855]	0.598±0.175 [0.508-0.688]	0.565±0.070 [0.529-0.601]
Specificity	0.861±0.039 [0.841-0.881]	0.858±0.113 [0.800-0.916]	0.827±0.080 [0.786-0.868]	0.899±0.062 [0.867-0.931]	0.649±0.192 [0.550-0.748]	0.855±0.023 [0.843-0.867]
MCC	0.556±0.113 [0.498-0.614]	0.657±0.083 [0.614-0.700]	0.325±0.162 [0.242-0.408]	0.685±0.119 [0.624-0.746]	0.262±0.163 [0.178-0.346]	0.429±0.091 [0.382-0.476]

(b)

	COG	COG$_{NC}$	COG$_{MCI}$	COG$_{DE}$	ADD	4-way
Accuracy	0.520±0.029 [0.484-0.556]	0.734±0.021 [0.708-0.760]	0.600±0.047 [0.542-0.658]	0.706±0.039 [0.658-0.754]	0.688±0.048 [0.628-0.748]	0.412±0.026 [0.380-0.444]
F-1	0.475±0.030 [0.438-0.512]	0.398±0.055 [0.330-0.466]	0.341±0.077 [0.245-0.437]	0.686±0.037 [0.640-0.732]	0.754±0.028 [0.719-0.789]	0.375±0.034 [0.333-0.417]
Sensitivity	0.479±0.029 [0.443-0.515]	0.360±0.091 [0.247-0.473]	0.432±0.146 [0.251-0.613]	0.644±0.067 [0.561-0.727]	0.952±0.030 [0.915-0.989]	0.412±0.026 [0.380-0.444]
Specificity	0.761±0.018 [0.739-0.783]	0.859±0.050 [0.797-0.921]	0.656±0.098 [0.534-0.778]	0.768±0.101 [0.643-0.893]	0.424±0.106 [0.292-0.556]	0.804±0.009 [0.793-0.815]
MCC	0.247±0.045 [0.191-0.303]	0.241±0.044 [0.186-0.296]	0.078±0.075 [-0.015-0.171]	0.421±0.083 [0.318-0.524]	0.444±0.082 [0.342-0.546]	0.236±0.044 [0.181-0.291]

(c)

	COG	COG$_{NC}$	COG$_{MCI}$	COG$_{DE}$	ADD	4-way
Accuracy	0.618±0.042 [0.566-0.670]	0.878±0.017 [0.857-0.899]	0.634±0.045 [0.578-0.690]	0.724±0.050 [0.662-0.786]	0.688±0.097 [0.568-0.808]	0.544±0.022 [0.517-0.571]
F-1	0.619±0.034 [0.577-0.661]	0.804±0.023 [0.775-0.833]	0.441±0.027 [0.407-0.475]	0.612±0.098 [0.490-0.734]	0.740±0.046 [0.683-0.797]	0.499±0.037 [0.453-0.545]
Sensitivity	0.675±0.024 [0.645-0.705]	1.000±0.000 [1.000-1.000]	0.576±0.054 [0.509-0.643]	0.448±0.100 [0.324-0.572]	0.872±0.111 [0.734-1.010]	0.544±0.022 [0.517-0.571]
Specificity	0.830±0.019 [0.806-0.854]	0.837±0.023 [0.808-0.866]	0.653±0.071 [0.565-0.741]	1.000±0.000 [1.000-1.000]	0.504±0.271 [0.168-0.840]	0.848±0.007 [0.839-0.857]
MCC	0.497±0.035 [0.454-0.540]	0.751±0.028 [0.716-0.786]	0.205±0.049 [0.144-0.266]	0.537±0.078 [0.440-0.634]	0.396±0.204 [0.143-0.649]	0.401±0.031 [0.363-0.439]

(d)

	COG	COG$_{NC}$	COG$_{MCI}$	COG$_{DE}$	ADD	4-way
Accuracy	0.632±0.071 [0.544-0.720]	0.874±0.039 [0.826-0.922]	0.650±0.068 [0.566-0.734]	0.740±0.053 [0.674-0.806]	0.680±0.100 [0.556-0.804]	0.558±0.061 [0.482-0.634]
F-1	0.631±0.070 [0.544-0.718]	0.802±0.051 [0.739-0.865]	0.448±0.092 [0.334-0.562]	0.642±0.095 [0.524-0.760]	0.746±0.053 [0.680-0.812]	0.505±0.065 [0.424-0.586]
Sensitivity	0.683±0.065 [0.602-0.764]	1.000±0.000 [1.000-1.000]	0.568±0.132 [0.404-0.732]	0.480±0.107 [0.347-0.613]	0.912±0.053 [0.846-0.978]	0.558±0.061 [0.482-0.634]
Specificity	0.836±0.031 [0.798-0.874]	0.832±0.052 [0.767-0.897]	0.677±0.071 [0.589-0.765]	1.000±0.000 [1.000-1.000]	0.448±0.238 [0.153-0.743]	0.853±0.020 [0.828-0.878]
MCC	0.510±0.087 [0.402-0.618]	0.748±0.064 [0.669-0.827]	0.221±0.145 [0.041-0.401]	0.562±0.083 [0.459-0.665]	0.407±0.167 [0.200-0.614]	0.409±0.075 [0.316-0.502]

Supplementary Table S6: Comparison of model performance with the neurologists. We randomly sampled 100 subjects from the NACC dataset. For each of the selected subject, we provided MRI scan along with a set of non-imaging features as specified in the supplementary material to 17 neurologists for them to review and make a prediction on one of the 4 possible categories, i.e., normal cognition (NC), mild cognitive impairment (MCI), Alzheimer's disease (AD), non-AD dementia (nADD). To make a head-to-head comparison, we also tested our MRI model, non-imaging model and fusion model on the same 100 selected subjects. We reported performance metrics, including accuracy, F-1, sensitivity, specificity and Matthew correlation coefficient (MCC) for various tasks as indicated by column names based on the predictions from (a) 17 neurologists, (b) the MRI model, (c) the non-imaging model and (d) the fusion model. The mean and standard deviation (std) from table (a) was calculated over all 17 neurologists, and the mean and std from the other tables was derived from 5-fold validation experiments. More specifically, the COG represents the full classification of NC, MCI and DE cases). In addition, we reported the performance of binary classification of NC vs. non-NC ("COG$_{NC}$" column), MCI vs. non-MCI ("COG$_{MCI}$" column) and DE vs. non-DE ("COG$_{DE}$" column). We also reported the model's performance in detecting AD from the dementied subjects within the "ADD columns. Lastly, we reported the 4-way classification of NC, MCI, AD, nADD ("4-way" column).

Supplementary Table S7

	Neuroradiologists	MR-only model
**Accuracy**	0.566±0.054 [0.516-0.616]	0.692±0.035 [0.649-0.735]
**F-1**	0.571±0.070 [0.506-0.636]	0.920±0.044 [0.865-0.975]
**Sensitivity**	0.589±0.122 [0.476-0.702]	0.464±0.090 [0.352-0.576]
**Specificity**	0.543±0.142 [0.412-0.674]	0.750±0.022 [0.723-0.777]
**MCC**	0.135±0.108 [0.035-0.235]	0.435±0.057 [0.364-0.506]

Supplementary Table S7: Comparison of model performance with the neuroradiologists. We randomly sampled 50 subjects from the NACC dataset. For each of the selected subject, we provided MRI scan along with a set of non-imaging features as specified in the supplementary material to 7 neuroradiologists for them to independently review and make a prediction on one of the 2 possible categories, i.e., Alzheimer's disease (AD) and non-AD dementia (nADD). We reported performance metrics, including accuracy, F-1, sensitivity, specificity and Matthew correlation coefficient (MCC) for this binary classification task by considering AD as positive samples.

Supplementary Table S8

Variable	COG_{NC} task AUC	COG_{NC} task AP	COG_{DE} task AUC	COG_{DE} task AP	ADD task AUC	ADD task AP
trailA	0.783	0.79	0.817	0.587	0.52	0.877
trailB	0.818	0.839	0.853	0.564	0.532	0.869
boston	0.791	0.762	0.825	0.59	0.569	0.887
digitB	0.725	0.719	0.753	0.458	0.533	0.891
digitBL	0.704	0.69	0.735	0.413	0.522	0.884
digitF	0.66	0.649	0.684	0.383	0.528	0.881
digitFL	0.632	0.624	0.654	0.329	0.54	0.885
animal	0.839	0.824	0.878	0.702	0.501	0.869
gds	0.647	0.633	0.6	0.275	0.608	0.895
lm_imm	0.872	0.86	0.907	0.722	0.638	0.913
lm_del	0.895	0.886	0.916	0.706	0.713	0.93
mmse	0.881	0.848	0.931	0.814	0.616	0.896
npiq_DEL	0.545	0.543	0.58	0.339	0.522	0.871
npiq_HALL	0.526	0.533	0.544	0.294	0.55	0.878
npiq_AGIT	0.597	0.574	0.628	0.357	0.501	0.86
npiq_DEPD	0.588	0.57	0.6	0.301	0.523	0.872
npiq_ANX	0.608	0.582	0.642	0.348	0.539	0.877
npiq_ELAT	0.513	0.527	0.516	0.246	0.508	0.868
npiq_APA	0.623	0.59	0.67	0.417	0.58	0.887
npiq_DISN	0.556	0.55	0.569	0.299	0.566	0.882
npiq_IRR	0.603	0.578	0.607	0.321	0.52	0.87
npiq_MOT	0.559	0.551	0.589	0.338	0.528	0.873
npiq_NITE	0.567	0.554	0.577	0.307	0.552	0.878
npiq_APP	0.575	0.561	0.595	0.32	0.541	0.875
faq_BILLS	0.794	0.742	0.928	0.79	0.511	0.859
faq_TAXES	0.807	0.762	0.936	0.801	0.522	0.872
faq_SHOPPING	0.733	0.676	0.88	0.752	0.538	0.875
faq_GAMES	0.706	0.673	0.841	0.689	0.571	0.879
faq_STOVE	0.632	0.602	0.73	0.55	0.53	0.878
faq_MEALPREP	0.709	0.677	0.853	0.71	0.521	0.885
faq_EVENTS	0.75	0.687	0.867	0.723	0.54	0.874
faq_PAYATTN	0.736	0.674	0.846	0.684	0.518	0.872
faq_REMDATES	0.82	0.756	0.925	0.776	0.527	0.871
faq_TRAVEL	0.781	0.716	0.908	0.766	0.501	0.864

Supplementary Table S8: Classification performance of each standalone neuropsychological test. To compare our machine learning models to sample thresholding of common neuropsychiatric tests, we measured the area under the curve (AUC) of the ROC curves, and averaged precision (AP) of the precision-recall curve for the COG_{NC}, the COG_{DE} and the ADD tasks, respectively on the NACC cohort. The AUC and AP were derived by simply thresholding on each of the raw neuropsychiatric test score. These results can be directly compared with the AUC and AP values that we reported within the ROC and PR plots of the Figure 3.

Supplementary Table S9

	AD	FTD	VD	DLB	PDD	Other
**NACC**	948	58	16	39	15	70
**OASIS**	193	4	2	7	1	13
**FHS**	17	2	3	3	0	1
**LBDSU**	0	0	0	0	13	0
**NIFD**	0	129	0	0	0	0
**ADNI**	369	0	0	0	0	0
**AIBL**	79	0	0	0	0	0
**Total**	1606	193	21	49	29	84

Supplementary Table S9: Distribution of demented cases. The number of nADD cases from the National Alzheimer's Coordinating Center (NACC), the Open Access Series of Imaging Studies (OASIS), the Framingham Heart Study (FHS), the Lewy Body Dementia Center for Excellence at Stanford University (LBDSU), the frontotemporal lobar degeneration neuroimaging initiative (NIFD), the Alzheimer's Disease Neuroimaging Initiative (ADNI) and the Australian Imaging, Biomarker & Lifestyle Flagship Study of Ageing (AIBL) datasets is illustrated by dementia subtypes, including Alzheimer's disease (AD), frontotemporal dementia (FTD), vascular dementia (VD), dementia with Lewy Bodies (DLB) and Parkinson disease dementia (PDD). The category of "Other" was used to describe the less-commonly encountered types of dementia with very low case numbers. This category includes conditions such as multisystem atrophy, progressive supranuclear palsy, Creutzfeld-Jakob disease, and Huntington's disease etc. Note that this table does not include the Parkinson's Progression Marker Initiative (PPMI) datasets, as the PPMI cohorts does not contain demented subjects.

I. Glossary of Tasks, Models, and Metrics

Diagnostic Tasks:

- **COG task:** Multiclass prediction of NC, MCI, and DE categories. May be further subdivided into the following subtasks:
  - **COG_{NC}:** The separation of persons with NC from those with MCI or DE.
  - **COG_{MCI}:** The separation of persons with MCI from those with NC or DE.
  - **COG_{DE}:** The separation of persons with DE from those with NC or MCI.

- **ADD task:** Separation of persons with AD from those with nADD given an initial diagnosis of DE.

- **4-way task:** Complete separation of NC, MCI, AD, and nADD cases. Accomplished by successive completion of the COG and the ADD tasks.

Model-Derived Cognitive Metrics

- **DEMO score:** “DEmentia MOdel” score. A continuous measure for overall cognitive status ranging from 0 (NC) to 1 (MCI) to 2 (DE). DEMO score thresholding enables completion of the COG task and its subtasks.

- **ALZ score:** “ALZheimer’s” score. A continuous measure from 0 (nADD) to 1 (AD) that corresponds with the probability that a person has Alzheimer’s disease dementia. ALZ score thresholding enables completion of the ADD task.

Model Types:

- **MRI-only model:** A convolutional neural network (CNN) that uses MRI scans and no other information to complete the COG and the ADD tasks.

- **Non-imaging model:** A traditional machine learning classifier that uses demographics, past medical history, neuropsychological testing, and functional assessments to complete the COG and the ADD tasks.

- **Fusion model:** A hybrid model composed of a CNN linked to a CatBoost model. The CNN portion computes the DEMO and the ALZ scores from MRI which are concatenated with non-imaging clinical variables. The CatBoost model then successively completes the COG and the ADD tasks.

II. Information Availability by Cohort

Below, we show a table summarizing and showing the comparison of non-imaging data variables across our 8 different study cohorts (further broken down by study data collection guidelines in the cohort). This table helps to clarify how more widely available non-imaging variables were included in our study.	**NACC** [4822]		**OASIS** [666]	**ADNI** [n = 1821]				**FHS** [n = 313]			**AIBL** [661]	**PPMI** [198]	**NIFD** [253]	**LBDSU** [182]	
	**UDS 3.0**	**UDS 2.0**	**UDS 2.0**	**ADNI 3**	**ADNI 2**	**ADNI GO**	**ADNI 1**	**FHS Gen 1**	**FHS Gen 2**	**FHS Gen 3**	**AIBL**	**PPMI**	**NIFD**	**Stanford**	**Study Inclusion**
**History**															
Age	Yes	Yes	Yes	Yes	Yes	Yes	Yes	Yes	Yes	Yes	Yes	Yes	Yes	Yes	*
Gender	Yes	Yes	Yes	Yes	Yes	Yes	Yes	Yes	Yes	Yes	Yes	Yes	Yes	Yes	*
Race	Yes	Yes	Yes	Yes	Yes	Yes	Yes	Yes	Yes	Yes	No	Yes	Yes	No	*
Hispanic/Latino	Yes	Yes	Yes	Yes	Yes	Yes	Yes	Yes	Yes	Yes	No	Yes	No	No	*
Years of Education	Yes	Yes	Yes	Yes	Yes	Yes	Yes	Yes	Yes	Yes	No	Yes	Yes	Yes	*
Marital Status	Yes	Yes	Limited	Yes	Yes	Yes	Yes	Yes	Yes	Yes	No	No	No	No	
Living Situation/Independence	Yes	Yes	Limited	Yes	Yes	Yes	Yes	No	No	No	No	No	No	No	
Other History	Limited	Limited	Limited	Limited	Limited	Limited	Limited				No	Limited	No		
**Past Medical History**															
Medical History - Cardiovascular	Yes	Yes	Yes	Yes	Yes	Yes	Yes	Yes	Yes	Yes	Yes	Yes	No		*
Medical History - Neurological	Yes	Yes	Yes	Yes	Yes	Yes	Yes	Yes	Yes	Yes	Yes	Yes	No		*
Medical History - Psychiatric	Yes	Yes	Yes	Yes	Yes	Yes	Yes	Yes	Yes	Yes	Yes	Yes	No		*
Smoking History	Yes	Yes	Yes	Yes	Yes	Yes	Yes	Yes	Yes	Yes	Yes	No	No	No	*
Alcohol History	Yes	Yes	Yes	Yes	Yes	Yes	Yes	Yes	Yes	Yes	No	No	No	No	*
**Medications**															
Medications	Yes	Yes	Limited	Yes	Yes	Yes	Yes	Yes	Yes	Yes	No	Yes	No	No	
**Family History**															
Family History of Dementia or Cognitive Impairment	Yes	Yes	Yes	Yes	Yes	Yes	Yes	Yes	Yes	Yes	No	Yes	No	No	*
**Vitals**															
Weight	Yes	Yes	Yes	Yes	Yes	Yes	Yes	Yes	Yes	Yes	No	Yes	No	No	
Height	Yes	Yes	Yes	Yes	Yes	Yes	Yes	Yes	Yes	Yes	No	Yes	No	No	
Blood Pressure	Yes	Yes	Yes	Yes	Yes	Yes	Yes	Yes	Yes	Yes	No	Yes	No	No	
Heart Rate	Yes	Yes	Yes	Yes	Yes	Yes	Yes	Yes	Yes	Yes	No	Yes	No	No	
Respiratory Rate	No	No	No	Yes	Yes	Yes	Yes				No	No	No		
Temperature	No	No	No	Yes	Yes	Yes	Yes	No	No	No	No	Yes	No	No	
**Neurological Exam**															
Gait	Yes	Yes	Limited	Yes	Yes	Yes	Yes	Yes	Yes	Yes	No	Yes	No	No	
Resting Tremor	Yes	Yes	Limited	Yes	Yes	Yes	Yes	No	Limited	Limited	No	Yes	No	No	
Bradykinesia	Yes	Yes	Limited	No	No	No	No	No	No	No	No	Yes	No	No	
Rigidity	Yes	Yes	Limited	No	No	No	No	No	Limited	Limited	No	Yes	No	No	
Other Neurological Exam	Yes	Yes	Limited	Limited	Limited	Limited	Limited	Limited	Limited	Limited	No	Yes	No	No	
**Clinical Diagnosis**															
Clinical Diagnosis	Yes	Yes	Yes	Yes	Yes	Yes	Yes	Yes	Yes	Yes	Yes	Yes	Yes	Yes	
**Labs/Imaging**															
APOE Genotyping	Yes	Yes	Limited	Yes	Yes	Yes	Yes	Limited	Yes	Yes	Yes	Limited	No	No	
Blood Studies	Yes	Yes	Limited	Yes	Yes	Yes	Yes	Yes	Yes	Yes	Yes	Yes	No	No	
CSF Studies	Yes	Yes	Limited	Yes	Yes	Yes	Yes	No	No	No	No	Yes	No	No	
FDG-PET	Yes	Yes	Limited	Yes	Yes	Yes	Yes	Limited	Limited	Limited	No	Yes	No	No	
Amyloid-PET/Tau-PET	Yes	Yes	Limited	Yes	Yes	Yes	Yes	Limited	Limited	Limited	No	Yes	No	No	
DAT Scan	No	No	No	No	No	No	No	No	No	No	No	Yes	No	No	
Neuropathology	Yes	Yes	Limited	Yes	Yes	Yes	Yes	Yes	Yes	Yes	No	No	No	No	
**Cognitive Measures**															
MMSE	No	Yes	Yes	Yes	Yes	Yes	Yes	Yes	Yes	No	Yes	No	Yes	No	*
MOCA	Yes	No	No	Yes	Yes	Yes	No	No	No	Yes	No	Yes	Yes	Yes	
American National Adult Reading Test (AMNART)	No	No	No	Yes	Yes	Yes	Yes	No	No	No	No	No	No	No	
Wide Range Achievement Test – Reading	No	No	No	No	No	No	No	Yes	Yes	Yes	No	No	No		
Craft Story 21 Recall Immediate Verbatim	Yes	No	No	No	No	No	No	No	No	No	No	No	No	No	
Craft Story 21 Recall Immediate Paraphrase	Yes	No	No	No	No	No	No	No	No	No	No	No	No	No	
Craft Story 21 Recall Delay Verbatim	Yes	No	No	No	No	No	No	No	No	No	No	No	No	No	
Craft Story 21 Recall Delay Paraphrase	Yes	No	No	No	No	No	No	No	No	No	No	No	No	No	
Logical Memory, Story A (WMS-R), Immediate Recall	No	Yes	Yes	Yes	Yes	Yes	Yes	Yes	Yes	Yes	Yes	No	No	No	*
Logical Memory, Story A (WMS-R) Delayed Recall	No	Yes	Yes	Yes	Yes	Yes	Yes	Yes	Yes	Yes	Yes	No	No	No	*
CERAD Word List Memory Task	No	No	No	No	No	No	No	No	No	Yes	No	No	No		
Rey Auditory Verbal Learning Test (AVLT)	No	No	No	Yes	Yes	Yes	Yes	No	No	No	No	No	No	No	
ADAS-Cog Word Recall & Recognition Test	No	No	No	Yes	Yes	Yes	Yes	No	No	No	No	No	No	No	
Hopkins Verbal Learning Test (HVLT)	No	No	No	No	No	No	No	No	No	No	No	Yes	No	No	
Benson Complex Figure Copy	Yes	No	No	No	No	No	No	No	No	No	No	No	Yes	No	
Benson Complex Figure Recall	Yes	No	No	No	No	No	No	No	No	No	No	No	Yes	No	
ADAS-Cog Construction Praxis Test	No	No	No	Yes	Yes	Yes	Yes	No	No	No	No	No	No	No	
Clock Drawing Test	es (MOC/	No	No	Yes	Yes	Yes	Yes	Yes	Yes	Yes	No	Yes	Yes	No	
Digit Span Test Forward	Limited	Yes	Yes	No	No	No	Yes	Yes	Yes	Yes	No	No	Yes	No	*
Number Span Test Forward	Yes	No	No	No	No	No	No	Yes	Yes	Yes	No	No	No	No	
Multilingual Naming Test (MINT)	Yes	No	No	Yes	No	No	No	No	No	No	No	No	No	No	
Boston Naming Test (BNT)	No	Yes	Yes	No	Yes	Yes	Yes	Yes	Yes	Yes	No	Yes	Yes	No	*
ADAS-Cog Naming Test	No	No	No	Yes	Yes	Yes	Yes	No	No	No	No	No	No	No	
Phonemic test, F words	Yes	No	No	Yes	Yes	Yes	No	Yes	Yes	Yes	No	Yes	No	No	*
Phonemic test, L words	Yes	No	No	No	No	No	No	No	No	No	No	No	No	No	
Phonemic test, D words	No	No	No	No	No	No	No	No	No	No	No	No	Yes	No	
Animals list generation	Yes	Yes	Yes	Yes	Yes	Yes	Yes	Yes	Yes	Yes	No	Yes	Yes	No	*
Vegetables list generation	Yes	Yes	Yes	No	No	No	Yes	No	No	No	No	Yes	No	No	
Trail making test part A	Yes	Yes	Yes	Yes	Yes	Yes	Yes	Yes	Yes	Yes	No	Yes	Yes	No	*
Digit Span Test Backward	Limited	Yes	Yes	No	No	No	Yes	Yes	Yes	Yes	No	No	Yes	No	*
Number Span Test Backward	Yes	No	No	No	No	No	No	Yes	Yes	Yes	No	No	No	No	
Trail making test part B	Yes	Yes	Yes	Yes	Yes	Yes	Yes	Yes	Yes	Yes	No	Yes	Yes	No	*
Victoria Stroop Test (VST)	No	No	No	No	No	No	No	No	No	Yes	No	No	No		
ADAS-Cog Number Cancellation Test	No	No	No	Yes	Yes	Yes	Yes	No	No	No	No	No	No	No	
WAIS-R Digit Symbol Substitution Test	Yes	Yes	Yes	No	No	No	Yes	Yes	Yes	Yes	No	No	No	No	
Cogstate Brief Battery	No	No	No	Yes	No	No	No	No	No	No	No	No	No	No	
**Other Measures**															
AD8 Screening	No	No	No	No	No	No	No			Yes	No	No	No		
Cognitive Change Index	No	No	No	Yes	Yes	No	No	No	No	No	No	No	No	No	
Everyday Cognition Questionnaire	No	No	No	Yes	Yes	Yes	No	No	No	No	No	No	No	No	
Geriatric Depression Scale (GDS)	Yes	Yes	Yes	Yes	Yes	Yes	Yes	No	No	No	No	Yes	Yes	No	*
Center for Epidemiological Studies - Depression (CES-D)	No	No	No	No	No	No	No	Yes	Yes	Yes	No	No	No		
State Trait Anxiety Inventory (STAI)	No	No	No	No	No	No	No	No	No	No	No	Yes	No	No	
Neuropsychiatric Inventory Questionnaire (NPI-Q)	Yes	Yes	Yes	Yes	Yes	Yes	Yes	No	No	No	No	No	Yes	No	*
Revised Self-Monitoring Scale	Yes	No	No	No	No	No	No	No	No	No	No	No	Yes	No	
Functional Assessment Questionnaire (FAQ)	Yes	Yes	Yes	Yes	Yes	Yes	Yes	No	No	No	No	No	Yes	No	*
Schwab & England Activities of Daily Living	No	No	No	No	No	No	No	Limited	Limited	Limited	No	Yes	Yes	No	
Clinical Dementia Rating (CDR)	Yes	Yes	Yes	Yes	Yes	Yes	Yes	No	No	No	Yes	No	Yes	No	
Unified Parkinson's Disease Rating Scale (MDS-UPDRS)	Yes	Yes	Limited	No	No	No	No	No	No	No	No	Yes	Yes	No	
Unified Huntington's Disease Rating Scale (UHDRS)	No	No	No	No	No	No	No	No	No	No	No	No	Yes,	No	
PSP Rating Scale	No	No	No	No	No	No	No	No	No	No	No	no	Yes	No	
Hachinski Ischemic Score	Yes	Yes	Limited	Yes	Yes	Yes	Yes	Yes	Yes	Yes	No	No	No	No	
Financial Capacity Instrument (FCI)	No	No	No	Yes	No	No	No	No	No	No	No	No	No	No	

III. Non-Imaging Features Used in Model Development

Demographics
- Age
- Gender
- Education

Medical History
- Family history of cognitive impairment
- History of heart attack/cardiac arrest
- History of atrial fibrillation
- History of angioplasty/endarterectomy/cardiac stenting
- History of cardiac bypass procedure
- History of pacemaker
- History of hypertension
- History of hypercholesterolemia
- History of heart failure
- History of other cardiovascular disease
- History of stroke
- History of transient ischemic attack
- History of seizures
- History of traumatic brain injury
- History of diabetes
- History of vitamin B12 deficiency
- History of thyroid disease
- History of urinary incontinence
- History of bowel incontinence
- History of depression within preceding two years
- History of depression greater than two years ago
- History of other psychiatric disorder
- History of alcohol use disorder
- Has smoked >100 cigarettes in life
- Total years smoking cigarettes
- Packets of cigarettes smoked per day
- History of other drug use

Neuropsychiatric Inventory
- Delusions
- Hallucinations
- Agitation/Aggression
- Dysphoria/Depression
- Anxiety
- Euphoria/Elation
- Apathy/Indifference
- Disinhibition
- Irritability/Lability
- Aberrant Motor Activity
- Nighttime Behavior
- Appetite/Eating

Neuropsychological Testing
- Trail Making Test Part A/B
- Boston Naming Test
- Digit span backward trials correct
- Digit span backward length
- Digit span forward trials correct
- Digit span forward length
- Animals
- Geriatric Depression Scale (GDS)
- Logical memory immediate recall
- Logical memory delayed recall
- Mini Mental State Exam (MMSE)

Functional Activities
- Paying Bills
- Assembling tax records
- Shopping alone
- Playing a game
- Meal preparation
- Keeping track of current events
- Paying attention to TV, books, or magazines
- Remembering dates
- Traveling and driving

IV. Neurologist Accounts of Diagnostic Approach

Abbreviations

- NP: Neuropsychology
- FAQ: Functional assessment questionnaire
- GDS: Geriatric depression scale
- NPI: Neuropsychiatric inventory
- MRI: Magnetic resonance imaging
- MMSE: Mini mental state examination
- WMH: White matter hyperintensity
- ADL: Activities of daily living
- IADL: Instrumental activities of daily living

Neurologist 1:
Demographic information was reviewed along with a summary of the subject's historical medical conditions, both active and inactive. Neuropsychological results featuring both raw and standardized scores (where available) were then reviewed, along with a FAQ, GDS and NPI total with sub-scores corresponding in time to the subject's cognitive assessments and stated age. Subjects with abnormal cognitive performance and elevated FAQ scores were potentially experiencing dementia. Functional ratings were considered in the context of potential progressive neurodegenerative disease as well as medical factors, substance abuse and overall burden of psychiatric symptoms. When specific cognitive domains appeared to be impaired out of proportion to others (such as episodic memory and low-frequency word-finding, etc.), the relative compatibility of the subject's cognitive profile with Alzheimer's disease was additionally considered. MRI was reviewed in the context of the above-described cognitive and functional impression. Particular attention was paid to the severity and distribution of any atrophy present, and for additional abnormalities that could alternatively explain or contribute to the subject's cognitive performance and functional status.

Neurologist 2:
The degree of cognitive impairment was first inferred from the raw cognitive assessment scores, as well as their associated Z-scores and percentiles. All subjects whose testing was within normal ranges received a label of NC. For all subjects, any cognitive domain score greater than 2 Z-scores below the mean or less than the 5th percentile was deemed to indicate cognitive impairment within that domain. If any cognitive impairment was present, the FAQ was used to distinguish between MCI and dementia: specifically, FAQ less than 5 qualified as MCI whereas FAQ greater than 5 was judged as dementia. For all cases of dementia, the subtype of dementia was inferred from the most prominent areas of brain atrophy, the most salient domains of cognitive impairment, evidence of vascular damage, and the size of the ventricle. Prominent features indicating AD included delayed memory impairment, the degree of medial temporal lobe atrophy and parietal atrophy. Prominent posterior parietal atrophy is also suggestive of AD. Otherwise, notable indicators of non-AD dementias included prominent executive dysfunction, mental behavior abnormalities, language disorders, asymmetric temporal lobe atrophy, frontal lobe atrophy, prominent white matter hyperintensities, and additional ischemic lesions such as those of the thalamus

and temporal lobe. There were several challenges worth noting. For instance, if FAQs are significantly reduced in cases of MCI, it is difficult to determine whether decreased FAQ is due to dementia or other physical, mental, or emotional factors. Broadly speaking, establishing a clear demarcation between MCI and dementia is challenging. Furthermore, when brain atrophy and cognitive decline are more global in nature, the task of subtyping the patient's dementia becomes more difficult.

Neurologist 3:
The participant's age, background history including family history, co-morbidities and cognitive assessments including MMSE were reviewed. This was followed by their functional assessment, GDS, and neuropsychiatric inventory. The degree and location of atrophy on MRI was then reviewed. If the participant had a normal cognitive and functional assessment with normal MRI, regardless of age, then this was labeled as normal. If the participant had a normal cognitive and functional assessment but was above 80 years with some mild generalized atrophy on MRI, then this was labeled as normal. If the patient had mildly impaired cognitive assessment with severe depression, and normal MRI, then this was also labeled normal. If the participant had mildly impaired cognitive assessment but no functional limitations with normal or mild atrophy on MRI, then this was labeled as mild cognitive impairment. The exception being if they had atrophy on MRI and were young (i.e., in their 50s). If the participant was noted to have impaired cognitive assessment with impaired functional assessment, with medial temporal or hippocampal atrophy out of proportion of the rest of the brain, then this was labeled AD. If the participant had impaired cognitive and functional assessment with significant vascular risk factors and small vessel disease on MRI, frontal prominent atrophy, or prominent neuropsychiatric inventory including delusions and hallucinations, this was labeled as non-ADD.

Neurologist 4:
The approach to these cases was akin to that which is taken with a patient presenting to the clinic. Though a personal history and physical evaluation were not available, the case sheets did provide most of the information necessary for the evaluation of a patient with underlying cognitive disorder. FAQ and NPI first gauged the severity of cognitive deficits and associated symptoms. Any patient who indicated dependency on others for functional activities solely due to cognitive difficulty was considered to have dementia. If the patient had mild cognitive symptoms, then they were diagnosed with MCI. If no symptoms were present and the patient was completely independent, then cognition was deemed to be age appropriate. Next, the GDS was used to assess for the presence of geriatric depression, which is a well-known confounder for cognitive disorder. In the case of depression, a patient's cognitive difficulty cannot be clearly resolved as occurring primarily or secondarily to another disease. After this, a full review of the patient's active medical problems was performed to assess alternative etiologies of cognitive decline such as stroke, TBI, seizures, thyroid disease, vitamin B12 deficiency, and substance abuse. If no such conditions were found, the cognitive disorder was considered to likely be secondary to Alzheimer's disease. Additional patient history, including age, gender, race, and family history was also reviewed for its diagnostic potential. Cognitive assessments were reviewed for patterns of deficiencies using both their z-score and percentile ratings. Significant abnormalities of memory testing both immediate and delayed recall would be highly suggestive of Alzheimer's disease in a symptomatic patient. Preserved memory function with either normal or abnormal language, processing, attention, and executive domains would be unusual for Alzheimer's disease. Lastly, the patient's brain MRI was reviewed for two reasons. 1) To rule

out structural causes of cognitive decline such as stroke, and 2) to look for classic atrophy patterns which may be suggestive of AD vs. non-ADD.

Neurologist 5:

The patient's clinical data was first reviewed. Extremes in education (e.g., less than completion of 9th grade) or age (eg >90 years old) were particularly notable. Thereafter, cognitive test scores were examined and double-checked to ensure that any normal scores were not accompanied by significant impairment in function- specifically, no FAQ scores >6. For any given patient, if cognition was normal and function intact, then the diagnosis was NC. Similarly, if function was intact per FAQ testing, but there were mild declines in cognitive test scores, then a diagnosis of MCI was made. Finally, if cognitive test scores were significantly low (greater than two Z-scores below the mean in at least 2 domains) and functional impairment present, then the patient was judged to have dementia. For those with dementia, the presence of a recent stroke or TBI was assessed. The scoring pattern on cognitive testing was also examined to judge its consistency with memory impairment. If there was evidence of dementia but no significant memory changes, then the other domains involved were considered, and a review of the NPI-Q for signs of hallucinations and disinhibition was completed. After checking this additional data, the brain MRI of all patients with dementia was reviewed. If imaging suggested an AD pattern of atrophy, then the diagnosis was AD. If the pattern was not suggestive of Alzheimer's, then the diagnosis was non-ADD. Naturally, there were certain exceptions to the above-outlined approach. Some patients demonstrated clear declines in cognition despite normal function per FAQ testing. Conversely, some patients exhibited only mild cognitive changes despite significantly elevated FAQ scores. For such patients with isolated functional impairment, additional efforts were made to look for signs of neuropsychiatric symptoms on the NPI or GDS, as these could feasibly explain functional impairment. Thus, patients with isolated functional impairment and high GDS or NPI scores were classified as MCI as opposed to dementia. Of note, for those with at least 9 years of education and an MMSE < 24, a low FAQ score was typically ignored, and dementia was diagnosed. For patients with particularly low levels of education (e.g., 3-5 years), functional testing was given relatively greater weight than cognitive testing. Various caveats should be considered. To begin, many of the subjects classified as MCI likely have prodromal AD pathology, and thus their brain MRI may have reflected an AD pattern of atrophy. Given that this project focused on AD at the stage of full clinical dementia, the brain MRI of those with an MCI diagnosis was not reviewed. MCI was thus taken to be a purely clinical diagnosis. Future studies may consider including MCI subjects, sub-classify them as amnestic vs. non-amnestic, and then have clinicians review brain MRIs to see whether they believe the subject may have prodromal AD. Additionally, it is quite common to have mixed pathology in the setting of dementia. For instance, recent studies suggest that approximately 50% of patients meeting criteria for Dementia with Lewy Bodies also have some AD pathology, and that pure Lewy Body disease was seen only in 39% of patients. Similarly, the co-occurrence of AD and vascular pathology is quite common. Future studies may want to include the possibility of classifying the subject as having co-occurring pathologies in the setting of AD.

Neurologist 6:

When approaching each case, demographic information, including age and education, were reviewed. Medical history and co-morbidities were also reviewed. Then the provided neuropsychological and psychiatric data was first reviewed along with the ADL scores. Significant functional impairment such as decreased ability to perform ADLs was useful in diagnosing dementia as well as distinguishing MCI from

dementia. Neuropsychological scores were then reviewed, as well as the FAQ, GDS, and NPI. Raw scores, standardized scores, and sub scores were reviewed for each patient. Different batteries on neuropsychological testing were also significantly considered. For example, profound executive dysfunction with relatively preserved memory function and lack of amnestic features would be more suggestive of Lewy Body Dementia or other non-AD dementia, whereas predominantly amnestic features being suggestive of AD. Patients with impaired performance on neuropsychological assessments were categorized into experiencing dementia versus MCI. As previously noted, ADL scores could be helpful in determining degree of cognitive impairment. Imaging was then reviewed. Patterns of atrophy were noted to assess for neurodegenerative disease type and disease burden. For example, parietal atrophy being more suggestive of atrophy, frontal-temporal atrophy more suggestive of frontal temporal dementia, and subcortical atrophy suggestive of vascular dementia. The volume loss/atrophy was also considered, with higher degrees of atrophy potentially correlating with greater clinical findings suggestive of cognitive impairment. The final diagnosis considered neuropsychological scores, imaging findings, and clinical history to assess if other medical comorbidities were contributing to cognitive impairment. There were some difficulties in making the diagnosis due to limitations in the presented data. It is difficult to determine from the provided information if ADL limitations occur secondarily to motor or cognitive impairment. Particularly in the case of non-ADDs such as Parkinson's disease, early limitations in ADLs may be due to motor impairment, whereas those who have progressed to Parkinson's disease dementia may score low on ADL assessments due to cognitive or executive dysfunction. Patients with good cognitive reserve may also perform average on neuropsychological testing despite significant executive impairment, and this may make pairing ADL assessment with neuropsychological assessment inadequate when determining dementia diagnoses. It is also important to note that there is often overlap of dementia pathologies. Notably, multiple dementias may account for difficult clinical presentations. Also, pathologic changes suggestive of multiple comorbid conditions are frequently noted on autopsy, as in the case of a patient with mixed PD/AD who is found to have both alpha-synucleinopathy and beta amyloid plaques.

Neurologist 7:
The primary driver in deciding the ratings was the cognitive test scores, which were first reviewed. If all the scores were average or above average (based on age and education adjusted z-scores), then the diagnosis was assigned as normal. If any test scores were below average or impaired, then the functional assessment scores were reviewed to determine if there was sufficient evidence for a functional impairment due to cognitive impairment, which would indicate dementia. If there was no functional impairment, then the diagnosis was assigned as MCI. If there was functional impairment that seemed reasonable in relation to the cognitive test impairments, then the diagnosis was dementia. For all demented cases, other information was used to determine AD vs non-ADD including pattern of cognitive impairment, type of functional impairments, NPI results, and MRI evidence of hippocampal atrophy out of proportion to global atrophy. Difficulties in rating occurred when there were average or above average cognitive test scores, indicating normal cognitive performance, but abnormalities on the functional rating or the NPI. Other difficulties arose when the cognitive test scores were only below average on one test, but not impaired, or when there was a mismatch between the type of cognitive test impairment and the type of functional impairment. Finally, in severely demented patients when all test scores were severely impaired and there was severe functional impairment, distinguishing between AD vs non-AD was more difficult.

Neurologist 8:
A review of the subject's demographic, clinical and cognitive measures followed by MRI was performed. The subject's education, age and MMSE were particularly considered while making diagnosis. If MMSE was below 25, then these subjects were generally diagnosed with dementia unless there were extenuating circumstances such as old age or poor level of education. Cognitive testing with poor recall, language involvement, slow processing speed and significantly low values on functional status were more indicative of MCI or AD. MRI was particularly useful in subjects whose depression and neuropsychiatric scores were elevated. If the MRI was normal and the subjects had depression and clinical testing was mildly affected, then the poor clinical scores were attributed to depression. If the subjects had mildly abnormal scores with some functional impairment and the MRI showed frontotemporal atrophy, then they were diagnosed with MCI. However, when the subjects were depressed and had poor scores on language and memory, then they were diagnosed with MCI. If the subject had disproportionately more language and behavior issues along with MMSE lower than 25, then the subjects were diagnosed with non-ADD. In some of these cases, MRI was confirmatory in providing evidence on frontotemporal atrophy. Also, if subjects had slow processing speed and executive function with decreased fluency and hallucinations and delusions, then they were diagnosed with non-AD. MRI was not necessarily indicative of diagnosis in these cases because most of them had generalized global atrophy, and posterior atrophy in some cases. If the subjects had MMSE below 25, and vascular risk factors with mild white matter disease on the MRI, then they were also diagnosed with non-ADD. All other cases with MMSE values lower than 25 were diagnosed with AD. In general, MCI diagnosis was not straightforward in depressed subjects or subjects with low education level.

Neurologist 9:
Initially, a demographic overview of the patient, including age, past medical history and MMSE was conducted. In the case of a normal MMSE, the likely diagnosis is NC. In that case, each MRI was reviewed to ensure no abnormalities. In the event of an MMSE between 27-30, MCI is considered if there is no significant other disease and the patient's MRI is unremarkable. For MMSE between 24-27, additional consideration was given to the total number of years of education and overall psychiatric disease burden, both of which can affect MMSE. As discussed, it was also assessed whether any MRI changes could be explained by prior medical history. In sum, a decision on the nature of the dementing process was based on a review of medical history, with particular attention paid to depression. Specific psychiatric symptoms such as severe hallucinations support a diagnosis other than Alzheimer's disease. The Boston Naming test and various memory assessments were also used in order to determine the particular subtype of dementia. In the case of dementia, consideration was given to imaging features which might explain cognitive impairment. If the dementia was explained by a finding on MRI consistent with a non-Alzheimer's process, then a non-ADD diagnosis was chosen. If there is no alternative explanation clinically and the MRI was supportive of AD, then the diagnosis was Alzheimer's disease.

Neurologist 10:
Clinical history was reviewed to determine if the subject met the criteria for cognitive impairment. Specifically, MMSE, FAQ and NPI scores were reviewed along with any evidence of psychiatric disease. If the MMSE scores were between 24-26, then other neuropsychological test scores were reviewed. If the Boston Naming test scores were between 14 and 20, then evidence of psychiatric risk factors that are typically related to depression were carefully reviewed. If clinical criteria for mild cognitive impairment

or dementia were noted, MRI was reviewed to identify patterns of atrophy. If atrophy was observed predominantly in the temporal and parietal lobes, then the diagnosis was deemed as AD. When global atrophy was present, and an unclear temporo-parietal predominance was seen, clinical history was used to guide the ultimate diagnosis.

Neurologist 11:
The subject's age along with MMSE were first reviewed as a quick screen of global cognition. Subsequently, FAQ scores were reviewed. If MMSE scores were low and the FAQ scores were above six, then the subject was considered as potentially having dementia rather than just MCI. Neuropsychological testing scores were then reviewed. Logical memory immediate and delayed recall scores were reviewed to see if the subject's scores were very impaired. If the scores remained low (e.g., generally in single digits), this indicated verbal learning or memory deficit. Scores from other cognitive domains were also reviewed to observe if there was a similar deficit or if these were relatively preserved. If verbal learning/memory were impaired significantly out of proportion to the other domains, then the diagnosis was assigned as AD. If other domains were significantly affected, and if the subject had many neuropsychiatric symptoms, then the diagnosis was assigned as non-ADD. MRI scans were also reviewed for most subjects when cognitive impairment was observed from the non-imaging data. Hippocampal atrophy observed on MRI reinforced the prior determinations that were based on cognitive scores. Challenges included not having information on the duration of symptoms or the approximate onset of symptoms (years prior to the assessments provided to the raters) and lack of visuospatial function test data.

Neurologist 12:
The images for each patient were viewed before reviewing their charts. This was done to prevent knowledge of the clinical history from confounding the image review. Examination of the MRI images in all 3 planes was performed to assess for evidence of focal or diffuse atrophy suggestive of a particular dementia diagnosis. Images were also reviewed for evidence of other pathological findings such as prior ischemic strokes, vascular disease, hydrocephalus, TBI, or other conditions. Review of MRIs was conducted without any volumetric analysis and estimated volume loss while accounting for age. After the patient's chart was reviewed to assess cognitive status. Attention was paid to the patient's cognitive function and, where abnormal, an attempt was made to determine whether cognitive dysfunction was global or limited only to certain domains. Imaging and cognitive study findings were correlated to reach the final diagnosis. If MRI and cognitive studies were normal, the diagnoses were normal. If the MRI showed signs of mild atrophy (diffuse or focal) or the cognitive studies showed mild cognitive decline, the diagnosis was MCI. If the MRI showed prominent diffuse atrophy or cognitive studies showed signs of decline across multiple domains, the diagnosis was AD. If the MRI showed focal atrophy or signs of atrophy consistent with ischemic strokes or frontotemporal dementia along with cognitive studies showing decline in limited cognitive domains only, the diagnosis was non-AD. Some cases had mixed features of AD and non-ADD and were classified as AD. Some cases with MCI had mild cognitive deficits consistent with early AD but were classified as MCI as MRI atrophy was mild and cognitive deficits were mild as well.

Neurologist 13:
The clinical information was reviewed to find evidence of vascular risk factors which may contribute to non-ADD. Cognitive test results such as MMSE were used for initial screening followed by review of the

performers in language, memory, and executive function domains. When scores were consistently lower in these domains, then AD was deemed as the diagnosis. In cases with near normal MMSE scores and lower scores in other domains, the diagnosis was not trivial and most often thought to be an MCI variant. Evidence of active psychiatric disorders and normal aging were also considered when diagnosing subjects with MCI. Finally, MRIs were helpful to confirm AD. However, in some cases, if the temporal lobe abnormalities were not present on MRI but other tests (e.g., neuropsychiatric inventory and ADLs) were supportive of dementia, AD was still deemed as the primary diagnosis.

Neurologist 14:
Subjects were diagnosed with normal cognition if they had high scores on their MMSE (29 or 30) and were within one standard deviation / z-score from the mean. Functionally, they had to be able to perform their ADLs and IADLs independently. Occasionally, this could be deceiving (e.g., a patient unable to perform ADLs but had very high scoring). These subjects were still classified as normal with the assumption that perhaps that they might be able to complete their ADLs because of other non-cognitive limitations (e.g., poor vision). Often, the MRI data did not significantly influence the classification of normal subjects because the clinical picture was reassuring. Subjects were classified as having MCI if they scored in the 24-28 range on their MMSE. Years of education were considered, and the z-scores were in the range of 1-2. Mild atrophy or evidence of white matter disease on the MRI were also observed in the cases diagnosed with MCI. Subjects were classified as AD if they had low MMSE scores and results from the other cognitive tests were consistently poor. If the subjects were cognitively impaired without many vascular risk factors or other signs (e.g., urinary incontinence), then they were deemed to have AD. Likewise, subjects with multiple high risk vascular features (e.g., atrial fibrillation, hypertension) and evidence of small vessel disease on MRI were classified as non-AD because vascular dementia was more likely. Evidence of delusions or hallucinations led to assigning non-ADD diagnosis on the subjects.

Neurologist 15:
The diagnoses of normal cognition, MCI, and dementia were initially considered primarily based on the existence of objective NP impairment. If at least one NP test result was below 1.5 SD or if two NP tests were below 1 SD, then these subjects were considered to have some level of cognitive impairment. Functional independence was considered when no FAQ items were scored greater than 1. Additional information was then reviewed including the MRI scans to determine the specific diagnosis. For AD, impaired delayed recall and medial temporal lobe atrophy provided supporting evidence. Some demented cases that did not have above typical AD characteristics but had atypical NP performance (e.g., more prominent non-memory impairment), parietal and (or) occipital lobes atrophy and no other contradictory evidence, were still diagnosed as AD (atypical type). For non-ADD, the subjects were diagnosed if they showed atypical NP performance, asymmetrical frontal or temporal atrophy on MRI that indicated FTD and related subtypes, or cerebrovascular disease, or hydrocephalus, etc. In rare cases, impaired FAQ was not considered associative with cognitive decline, e.g., due to severe depression or psychiatric disorders. These cases were not assigned to have dementia. In other rare cases, subjects whose FAQ was normal but had severe language problems and prominent temporal lobe atrophy were diagnosed as non-ADD as they may have primary progressive aphasia. The challenges during diagnosis included (1) lacking medical history (onset characteristics and course of disease) and data for neurological signs; (2) No NP data for

visuospatial performance; (3) No unanimously accepted operational definition for objective NP impairment and functional independency.

Neurologist 16:
The subject's history including age, gender, education level, past disease history, family history and other medical information was reviewed. This was followed by review of the cognitive assessments, FAQ, GDS and NPI-Q to determine if the subject had MCI or dementia. For extremely poor scores, dementia was deemed as the diagnosis. The AD cases were differentiated from non-ADD cases based on the nature of cognitive impairment and the condition of the combined neuropsychiatric symptoms. Additionally, if the MRI indicated an atrophy pattern within the medial temporal or parietal lobes, the subject was diagnosed with AD. If there was asymmetric frontotemporal lobe atrophy or significant cerebrovascular disease, then non-ADD was considered as the diagnosis.

Neurologist 17:
The subject was considered to have dementia if there were significant deficits on cognitive testing and functional impairments in more than one domain. Based on the age, the overall brain volume was examined on the MRI, followed by review of hippocampus/medial temporal lobe volume. The imaging was then assessed for WMD and other abnormalities. AD was selected as the diagnosis if there was a consistent pattern of atrophy and the history negative for prominent delusions/hallucinations. Non-ADD was chosen as the diagnosis if there was a high burden of WMD and/or prominent delusions/hallucinations. If there was WMD and disproportionate hippocampal/medial atrophy, then cognitive test results were used to determine if the pattern correlated with AD vs non-ADD; AD was selected as the diagnosis if there was preserved immediate recall/registration and poor delayed recall, and non-ADD as the diagnosis if the registration was very poor. The subject was considered to have MCI if there were deficits on cognitive testing and functional impairment in one domain. If severe abnormalities were identified on imaging in terms of WMD or atrophy pattern, then the diagnosis was updated to be either AD or non-ADD based on prior descriptors. The subject was considered to be NC if cognitive testing showed results within the normal ranges and was independent in all functional domains. If severe abnormalities were identified on imaging in terms of WMD or atrophy pattern, and if the cognitive testing results were lower than expected for the subject's level of education, then the diagnosis was updated to MCI.

Summary of the neurologist approach to the ratings
Collectively, these perspectives speak to the importance of an integrated approach to dementia diagnosis in which distinct modes of data are reconciled prior to an ultimate classification of disease status. Assessment of demographic and medical history were commonly employed to rule-out confounding conditions leading to cognitive decline, or to suggest specific symptomologies consistent with dementia subtypes. Relatedly, neurocognitive, and functional testing allowed clinicians to not only triage subjects by their degree of impairment, but also to delve into domain-specific declines in cognition that are important in differentiating various forms of dementia.

Most commonly, MRI was utilized as a confirmatory test of the initial clinical impression. Spatial patterns of atrophy were highly informative during neuroimaging reviews, with disproportionate volume

loss in the medial temporal and parietal lobes often cited as suggesting AD. Asymmetric atrophy outside of these regions, as well as ischemic lesions and excessive ventricular enlargement were typically judged to represent various non-ADDs.

Collectively, these perspectives speak to the importance of an integrated approach to dementia diagnosis in which distinct modes of data are reconciled prior to an ultimate classification of disease status.

V. Neuroradiologist Accounts of Diagnostic Approach

Neuroradiologist 1:

MRIs were initially screened to identify subjective evidence of any abnormality. Given that the task at hand was to discriminate between AD and non-ADD, careful attention was paid to potential patterns of volume loss--particularly within the bilateral hippocampi. If there was relatively symmetric severe hippocampal volume loss bilaterally, AD was typically chosen. Non-AD was typically diagnosed if the hippocampi were relatively spared and any of the following conditions were met: 1) notable asymmetry to volume loss (this is often observed in frontotemporal dementia or corticobasal degeneration) 2) disproportionate volume loss involving structures outside of the temporal and parietal lobes (e.g., frontal lobe, occipital lobe, brainstem, or cerebellum) 3) ventricle enlargement clearly discordant with the degree of cerebral volume loss (thus indicating possible normal pressure hydrocephalus). Among the challenges encountered in this study was the difficulty of providing diagnosis without having a consensus approach between radiologists. Additionally, limiting reads to T1-weighted pre-contrast sequences somewhat limited the assessment of vascular dementia. Additionally, there was nonuniform alignment of some sequences, which needed multiplanar reformats within Slicer to achieve uniform alignment.

Neuroradiologist 2:

A quick scan of the entire MRI was performed to check for motion or any other quality issues, and the presence of infarcts and extensive small vessel disease to suggest vascular dementia, followed by an initial global assessment of atrophy. The approach started with observing the ventricles and the corpus callosum. It was relatively easier to assess sulcal enlargement than parenchymal atrophy. An assessment was also made for presence of disproportionate ventriculomegaly to suggest normal pressure hydrocephalus. It was challenging to grade the amygdala, hippocampus, and parahippocampus separately and they were largely graded identically. Grading for parietal lobe atrophy was difficult because the parietal lobes, especially the postcentral gyri and superior parietal lobules often look smaller than the rest of the brain in older subjects. Therefore, unless severe, parietal lobe atrophy was downgraded by 1 level i.e., the same appearance in the frontal lobe that would be graded moderate for the parietal lobes would be graded mild, etc. AD dementia was called when there was predominant parietal and medial temporal atrophy. If there was severe frontal or occipital atrophy or global severe temporal atrophy, then those cases were diagnosed with non-AD dementia even if there was parietal and medial temporal atrophy. It is recognized that this method would likely miss posterior cortical variant of AD.

Neuroradiologist 3:
All MRI scans were reviewed to determine whether atrophy involved the hippocampus/medial temporal lobe, entorhinal cortex, and parietal lobes. If atrophy was present in these areas, the diagnosis was assigned as AD. Alternatively, non-ADD was diagnosed if extensive small vessel changes (suggesting vascular disease) were present, or if significant atrophy was observed in the frontal lobe or diffusely throughout the brain. The major challenges encountered included image quality or completeness. For instance, certain scans featured significant patient motion, or were otherwise missing certain sequences. Also, numerous cases had imaging features that overlapped between AD and non-ADD, thus making these difficult to definitively identify. For these patients, judgement between the two types of dementia was very subjective.

Neuroradiologist 4:
The medial temporal lobe on the coronal reformats were first reviewed for atrophy disproportionate to other brain regions. If present, then the axial, coronal, and sagittal reformats were reviewed to see if the atrophy also involved the anterior temporal lobes. In most instances, there was correlation between the degree of medial and anterior temporal lobe atrophy. Subsequently, axial reformats were reviewed to see if there was parietal lobe atrophy, the axial and coronal reformats to see if there was frontal lobe atrophy, and the coronal reformats to see if there was occipital lobe atrophy. When assessing for ventricular enlargement, the size of the ventricles was always compared to the sulci looking for disproportionate ventricular enlargement. Corpus callosum atrophy was evaluated using the sagittal reformats. AD was selected as the diagnosis for cases with both parietal and temporal lobe atrophy or only parietal lobe atrophy. Non-ADD was selected as the diagnosis for the other cases. "Mild" was chosen for subtle findings, "severe" was chosen for extreme findings such as "knife-like" gyri, and "moderate" was chosen for those findings that fell in between. Encountered difficulties including identifying regional predominant volume loss in those patients with superimposed generalized atrophy, segmenting the temporal and frontal lobes, and downloading/uploading individual datasets into 3D slicer.

Neuroradiologist 5:
The overall approach was to initially review the MRI scans to exclude the presence of multiple infarcts and observe easily identifiable atrophy patterns in the entire brain. For example, initial assessment focused on whether frontal and anterior temporal versus parietal and medial temporal volume loss was dominant and easily identifiable. The second stage of assessment involved a more detailed sub-analysis of each region and grading of severity. AD diagnosis was assigned when atrophy was observed predominantly within the parietal and medial temporal lobes or when the frontal lobe involvement was less than or commensurate with parietal and temporal lobes. Diagnosis of non-AD dementia was assigned in any pattern differing from this including frontal, anterior temporal, or occipital predominant involvement as well as enlarged ventricles or multiple infarcts. The size of the ventricles with respect to that of the sulci was compared when assessing ventricular size.

Neuroradiologist 6:
Regional atrophy was assessed by observing the size of the gyri and the degree of sulcal widening in different brain regions using the axial, sagittal, and coronal planes. Ventricular size was also assessed. Cases with disproportionate atrophy of the medial temporal lobes/hippocampus, in the absence of severe anterior temporal atrophy, were classified as AD. Cases with disproportionate parietal atrophy (with or

without medial temporal atrophy) in patients younger than 65 years of age were also classified as early onset AD. Patterns consistent with posterior cortical atrophy/visual variant of Alzheimer's disease were also investigated by looking for parietal +/- parieto-occipital and posterior temporal lobe predominant atrophy. All other patterns of atrophy were classified as non-ADD, such as disproportionately severe anterior temporal atrophy. Cases with no-to-minimal atrophy and cases without clear regional predominance were challenging to classify. Cases with medial temporal atrophy along with some degree of atrophy elsewhere in the temporal and/or frontal lobes were also a challenge.

Neuroradiologist 7:
The hippocampus and medial temporal lobes were first evaluated. Specific attention was paid to volume loss in these structures and additional lobes of the brain, and to whether changes in the bilateral cerebral hemispheres were symmetrical. If the volume in each brain lobe decreased proportionally, with dominant volume reduction in the hippocampal and/or parietal lobes, then the subject was diagnosed with AD. If the brain lobes decreased in volume disproportionately, specifically within the temporal pole, frontal, and occipital lobes, or if bilateral asymmetry was obvious, then the subjects were diagnosed with non-ADD. Ischemic changes were often observed. If during the evaluation process, cerebrovascular disease was obvious, then vascular dementia was considered a possibility. In such cases, the lack of obvious volume loss or the presence of proportional volume reduction in each lobe led to the diagnosis of vascular dementia (ie. non-ADD). However, if ischemic changes were noted in combination with prominent volume loss within the medial temporal or parietal lobes, a mixed AD/vascular dementia pathology was favored, and the subject was classified as AD overall.

Summary of the neuroradiologist approach to the ratings
The neuroradiologists' approach to dementia classification was predominantly informed by the distribution of atrophic changes relative to global brain atrophy. Expectedly, disproportionate volume loss within temporal lobe structures and the parietal lobe was judged to be consistent with AD, whereas such changes outside of these regions was generally deemed to represent non-ADD. Additional features commonly suggesting non-ADD included the presence of past infarcts as well as excessive ventricular enlargement.

VI. Data to Clinicians and Diagnostic Criteria

Section I: Data provided to the neurologists

In our expert-level validation, we gave our panel of neurologists a random subset of 100 NACC participants. We aimed to simulate the full span of assessment material available to a practicing physician. The example of a case presentation to a neurologist, including all the clinical non-imaging data features, is provided below.

Furthermore, we included a description of clinical data fields to the neurologists participating in our clinician vs. model comparison. The document below is what was provided to neurologists as an instructional form for further information on each of the clinical non-imaging data features.

After the neurologists reviewed the cases and the clinical data field descriptions, they were asked to give a diagnosis label for each case (either normal cognition, mild cognitive impairment, Alzheimer's disease dementia, or non-Alzheimer's disease dementia).

Case Example

**HISTORY**	
Age	78 years
Gender	Female
Race	White
Hispanic/Latino	Yes
Education	20 years
**PAST MEDICAL HISTORY**	
Heart attack/cardiac arrest	No
Atrial fibrillation	No
Angioplasty/endarterectomy/stent	No
Cardiac bypass procedure	No
Pacemaker and/or defibrillator	No
Congestive heart failure	No
Other cardiovascular disease	No
Stroke	No
Transient ischemic attack (TIA)	No
Seizures	No
Traumatic brain injury (TBI)	No
Hypertension	Yes, remote/inactive

Hypercholesterolemia	No
Diabetes	No
B12 deficiency	No
Thyroid disease	No
Incontinence (urinary)	No
Incontinence (bowel)	No
Depression	No
Other psychiatric disorder	No
Alcohol abuse	No
Smoking	No
Other substance abuse	No

FAMILY HISTORY

Family history of cognitive impairment (1° family)	No

COGNITIVE ASSESSMENT WITH NORMS (Z-score, percentile)

Global - MMSE	25/30 (-3.02, 0.13)
Memory – Logical Memory Immediate Recall (story units)	4 (-2.53, 0.57)
Memory – Logical Memory Delayed Recall (story units)	5 (unknown, unknown)
Language – Boston Naming Test	20/30 (unknown, unknown)
Language - Animal Words in 60 sec	19 (-0.57, 28.56)
Language - F Words in 60 sec	16 (unknown, unknown)
Processing Speed - Trails A (sec)	123 (-6.51, 0.0)
Attention - Forward Digit Span (trials correct)	8 (-0.72, 23.7)
Executive Function - Backward Digit Span (trials correct)	5 (-1.13, 12.85)
Executive Function - Trails B (sec)	300 (-5.13, 0.0)

FUNCTIONAL ASSESSMENT

Functional Activities Questionnaire (FAQ) - Total	0/30
FAQ - Bills	0 (Normal)
FAQ - Taxes	0 (Normal)
FAQ - Shopping	0 (Normal)
FAQ - Games	0 (Normal)
FAQ - Stove	0 (Normal)
FAQ - Meals	0 (Normal)
FAQ - Current Events	0 (Normal)
FAQ - Paying Attention	0 (Normal)
FAQ - Remembering Dates	0 (Normal)
FAQ - Travel	0 (Normal)

OTHER ASSESSMENT

Geriatric Depression Scale (GDS)	2/15
Neuropsychiatric Inventory (NPI-Q) - Total	6/36
NPI-Q - Delusions	0 (No)
NPI-Q - Hallucinations	0 (No)
NPI-Q - Agitation/Aggression	0 (No)
NPI-Q - Depression/Dysphoria	0 (No)
NPI-Q - Anxiety	0 (No)
NPI-Q - Elation/Euphoria	3 (Yes, severe)
NPI-Q - Apathy/Indifference	0 (No)
NPI-Q - Disinhibition	0 (No)
NPI-Q - Irritability/Lability	0 (No)
NPI-Q - Motor Disturbance	0 (No)
NPI-Q - Nighttime Behaviors	0 (No)
NPI-Q - Appetite/Eating	3 (Yes, severe)

Description of Clinical Data Fields

HISTORY

- Age
- Gender
- Race: There are 6 categories for race: White, Black or African American, American Indian or Alaska Native, Hawaiian or Pacific Islander, Asian, or Multiracial.
- Ethnicity: Hispanic/Latino
- Years of Education: Reported as 12 years for high school or GRE, 16 years for bachelor's degree, 18 years for master's degree, 20 years for doctoral degree

PAST MEDICAL HISTORY

- Completed by clinician following interview with subject and informant.
- Scoring:
  - No - not indicated by information
  - Yes, recent/active - occurred within last year or requires active management
  - Yes, remote/inactive - occurred in past (> 1 year ago) but was resolved or no treatment underway
  - Unknown - insufficient information OR information not collected
- Medical conditions:
  - Heart attack / cardiac arrest
  - Atrial fibrillation
  - Angioplasty / endarterectomy / stent
  - Cardiac bypass procedure

    - Pacemaker and/or defibrillator
        - For some subjects, question may be asked about pacemaker only
    - Congestive heart failure
    - Other cardiovascular disease
    - Stroke
    - Transient ischemic attack (TIA)
    - Seizures
    - Traumatic brain injury (TBI)
    - Diabetes
    - Hypertension
    - Hypercholesterolemia
    - B12 deficiency
    - Thyroid disease
    - Incontinence (urinary)
    - Incontinence (bowel)
    - Alcohol abuse: clinically significant impairment occurring over a 12-month period manifested in work, driving, legal, or social areas
    - Other abused/illicit substances: clinically significant impairment occurring over a 12-month period manifested work, driving, legal, or social areas
    - Other psychiatric disorder: psychiatric disorders other than depression
- Special Cases:
    - Depression: Reported as (1) Yes, active in last 2 years, (2) Yes, episodes more than 2 years ago, or (3) No
    - Smoking: Yes - if the subject has smoked more than 100 cigarettes in her/his life. No - if not.
        - If yes, total years smoked and average number of packs smoked per year reported.

FAMILY HISTORY

- Family history: Yes - if the subject had at least one first-degree relative (father, mother, sibling) with cognitive impairment. No - if not.

COGNITIVE ASSESSMENT WITH NORMS

- Note #1: Cognitive Assessment scores were taken from the closest visit to the MRI scan (within 6 months of MRI scan)
- Note #2: We provided norms for the neuropsych testing (where available), that gives an estimate of performance based on sex, age, and education. Unfortunately, norms were not available for the Boston Naming Test, the F Words, or for some of the Logical Memory Delayed Recall. The norms are given in the form of (Z-scores, percentiles).

- Mini-Mental State Exam (MMSE)[1]: A brief, quantitative measure of cognitive status in adults. Total score reported out of 30.
- Logical Memory[2]: This is the Logical Memory IA and IIA Test from Wechsler Memory Scale-Revised (WMS-R). Assesses recall of an oral story (immediate recall) and recall of the same story after a 30-minute delay (delayed recall). Total story units that can be recalled is 25.
- Boston Naming Test[3]: A test of naming where subjects are shown drawings of objects and asked to name them. Total score is out of 30.
- Animal Words: Total number of animals named in 60 seconds. Category fluency.
- F Words: Total number of words beginning with letter 'F' in 60 seconds. Verbal fluency.
- Trails Making Test[4]:
    - Trails A: A test where subjects draw lines connecting consecutive numbers. Total number of seconds to complete is reported, with 150 sec being the maximum time allotted.
    - Trails B: A test where subjects draw lines connecting numbers and letters in alternating progressive sequence. Total number of seconds to complete is reported, with 300 sec being the maximum time allotted.
- Digit Span: From Wechsler Adult Intelligence Scale-Revised (WAIS-R).[5]
    - Forward: A test where subjects repeat numbers spoken by the examiner. Reports total number of trials correct prior to two consecutive errors at the same digit length. Maximum 12 trials.
    - Backward: A test where subjects repeat numbers in the reverse order of that presented. Reports total number of trials correct prior to two consecutive errors at the same digit length. Maximum 12 trials.

FUNCTIONAL ASSESSMENT

- Functional Activities Questionnaire (FAQ)[6]: Completed by clinician based on information from informant. Informants answered based on if subjects had difficulty or needed help with the following items in the past 4 weeks.
    - Items:
        1. Bills – Writing checks, paying bills, or balancing a checkbook
        2. Taxes – Assembling tax records, business affairs, or other papers
        3. Shopping – Shopping alone for clothes, household necessities, or groceries
        4. Games – Playing a game of skill such as bridge or chess, working on a hobby
        5. Stove – Heating water, making a cup of coffee, turning off the stove
        6. Meals – Preparing a balanced meal
        7. Current Events – Keeping track of current events
        8. Paying Attention – Paying attention to and understanding a TV program, book, or magazine

9. Remembering Dates – Remembering appointments, family occasions, holidays, medications
10. Travel – Traveling out of the neighborhood, driving, or arranging to take public transportation

- Scoring: 0 (Normal), 1 (Has difficulty, but does by self), 2 (Requires assistance), or 3 (Dependent).
    - Another option is Not applicable, (e.g., never did).
    - Total score out of 30.

OTHER ASSESSMENTS

- Geriatric Depression Scale shortened (GDS-S)[7]: Completed by clinician based on subject response. Subjects answered yes or no based on how they have been feeling in the past week.
    - Items:
        1. Are you basically satisfied with your life?
        2. Have you dropped many of your activities and interests?
        3. Do you feel that your life is empty?
        4. Do you often get bored?
        5. Are you in good spirits most of the time?
        6. Are you afraid that something bad is going to happen to you?
        7. Do you feel happy most of the time?
        8. Do you often feel helpless?
        9. Do you prefer to stay at home, rather than going out and doing new things?
        10. Do you feel you have more problems with memory than most?
        11. Do you think it is wonderful to be alive now?
        12. Do you feel pretty worthless the way you are now?
        13. Do you feel full of energy?
        14. Do you feel that your situation is hopeless?
        15. Do you think that most people are better off than you are?
    - Scoring: 1 = Yes (#2-4, 6, 8-10, 12, 14-15), No (#1, 5, 7, 11, 13)
        - 0 = otherwise
        - Total score out of 15
- Neuropsychiatric Inventory (NPI-Q)[8]: Completed by clinician based on information from informant. Informants answered based on changes that have occurred since the subject first began to experience memory/cognitive problems. If yes, severity was also assessed (how it affects the subject).
    - Items:

1. Delusions — Does the patient have false beliefs, such as thinking that others are stealing from him/her or planning to harm him/her in some way?
2. Hallucinations — Does the patient have hallucinations such as false visions or voices? Does he or she seem to hear or see things that are not present?
3. Agitation/aggression — Is the patient resistive to help from others at times, or hard to handle?
4. Depression/dysphoria — Does the patient seem sad or say that he/she is depressed?
5. Anxiety — Does the patient become upset when separated from you? Does he/she have any other signs of nervousness such as shortness of breath, sighing, being unable to relax, or feeling excessively tense?
6. Elation/euphoria — Does the patient appear to feel too good or act excessively happy?
7. Apathy/indifference — Does the patient seem less interested in his/her usual activities or in the activities and plans of others?
8. Disinhibition — Does the patient seem to act impulsively, for example, talking to strangers as if he/she knows them, or saying things that may hurt people's feelings?
9. Irritability/lability — Is the patient impatient and cranky? Does he/she have difficulty coping with delays or waiting for planned activities?
10. Motor disturbance — Does the patient engage in repetitive activities such as pacing around the house, handling buttons, wrapping string, or doing other things repeatedly?
11. Nighttime behaviors — Does the patient awaken you during the night, rise too early in the morning, or take excessive naps during the day?
12. Appetite/eating — Has the patient lost or gained weight, or had a change in the type of food he/she likes?

- Scoring: 0 (No), 1 (Yes, mild), 2 (Yes, moderate), 3 (Yes, severe)
    - Total score out of 36
    - Yes - symptoms present in last month
    - No - otherwise
    - Mild - noticeable, but not a significant change
    - Moderate - significant, but not a dramatic change
    - Severe - very marked or prominent, a dramatic change

Section II: Data provided to the neuroradiologists

For our expert-level validation, we gave neuroradiologists 50 randomly sampled cases from the NACC dataset. Below, we provide a template questionnaire that was given to neuroradiologists. All neuroradiologists were provided with 1.5T T1-weighted MRIs for each subject, along with age and gender. The neuroradiologists filled out one online questionnaire for each case they reviewed and were asked to give a dementia diagnosis of Alzheimer's disease dementia and non-Alzheimer's disease dementia based on impression.

Neuroradiologist Questionnaire

1) Anterior Temporal Lobe
    a) Is there atrophy on the Anterior Temporal Lobe?
        i) Yes
        ii) No
    b) Left amygdala
        i) None (0)
        ii) Mild (1)
        iii) Moderate (2)
        iv) Severe (3)
        v) Cannot assess
    c) Right amygdala
        i) None (0)
        ii) Mild (1)
        iii) Moderate (2)
        iv) Severe (3)
        v) Cannot assess
    d) Left Anterior Temporal Lobe-Medial Part
        i) None (0)
        ii) Mild (1)
        iii) Moderate (2)
        iv) Severe (3)
        v) Cannot assess
    e) Right Anterior Temporal Lobe-Medial Part:
        i) None (0)
        ii) Mild (1)
        iii) Moderate (2)
        iv) Severe (3)
        v) Cannot assess
    f) Left Anterior Temporal Lobe-Lateral Part

i) None (0)
ii) Mild (1)
iii) Moderate (2)
iv) Severe (3)
v) Cannot assess

g) Right Anterior Temporal Lobe-Lateral Part
i) None (0)
ii) Mild (1)
iii) Moderate (2)
iv) Severe (3)
v) Cannot assess

2) Medial Temporal Lobe
a) Is there Medial Temporal Lobe atrophy?
i) Yes
ii) No

b) Left Hippocampus
i) None (0)
ii) Mild (1)
iii) Moderate (2)
iv) Severe (3)
v) Cannot assess

c) Right Hippocampus
i) None (0)
ii) Mild (1)
iii) Moderate (2)
iv) Severe (3)
v) Cannot assess

d) Left Parahippocampus
i) None (0)
ii) Mild (1)
iii) Moderate (2)
iv) Severe (3)
v) Cannot assess

e) Right Parahippocampus
i) None (0)
ii) Mild (1)
iii) Moderate (2)
iv) Severe (3)
v) Cannot assess

3) Parietal Lobe

a) Is there parietal lobe atrophy?
   i) Yes
   ii) No
b) Left Parietal Lobe
   i) None (0)
   ii) Mild (1)
   iii) Moderate (2)
   iv) Severe (3)
   v) Cannot assess
c) Right Parietal Lobe
   i) None (0)
   ii) Mild (1)
   iii) Moderate (2)
   iv) Severe (3)
   v) Cannot assess

4) Frontal Lobe
   a) Is there frontal lobe atrophy?
      i) Yes
      ii) No
   b) Left Orbitofrontal
      i) None (0)
      ii) Mild (1)
      iii) Moderate (2)
      iv) Severe (3)
      v) Cannot assess
   c) Right Orbitofrontal
      i) None (0)
      ii) Mild (1)
      iii) Moderate (2)
      iv) Severe (3)
      v) Cannot assess
   d) Left Dorsolateral
      i) None (0)
      ii) Mild (1)
      iii) Moderate (2)
      iv) Severe (3)
      v) Cannot assess
   e) Right Dorsolateral
      i) None (0)
      ii) Mild (1)

iii) Moderate (2)
iv) Severe (3)
v) Cannot assess

f) Left Superior
   i) None (0)
   ii) Mild (1)
   iii) Moderate (2)
   iv) Severe (3)
   v) Cannot assess

g) Right Superior
   i) None (0)
   ii) Mild (1)
   iii) Moderate (2)
   iv) Severe (3)
   v) Cannot assess

5) Occipital Lobe
   a) Is there occipital lobe atrophy?
      i) Yes
      ii) No
   b) Left Occipital Lobe
      i) None (0)
      ii) Mild (1)
      iii) Moderate (2)
      iv) Severe (3)
      v) Cannot assess
   c) Right Occipital Lobe
      i) None (0)
      ii) Mild (1)
      iii) Moderate (2)
      iv) Severe (3)
      v) Cannot assess

6) Ventricular Enlargement
   a) Is there any evidence of ventricular enlargement?
      i) Yes
      ii) No
   b) Left Lateral Ventricle-Temporal Horn
      i) None (0)
      ii) Mild (1)
      iii) Moderate (2)
      iv) Severe (3)

    v) Cannot assess

c) Right Lateral Ventricle-Temporal Horn
    i) None (0)
    ii) Mild (1)
    iii) Moderate (2)
    iv) Severe (3)
    v) Cannot assess

d) Left Lateral Ventricle-Excluding Temporal Horn
    i) None (0)
    ii) Mild (1)
    iii) Moderate (2)
    iv) Severe (3)
    v) Cannot assess

e) Right Lateral Ventricle-Excluding Temporal Horn
    i) None (0)
    ii) Mild (1)
    iii) Moderate (2)
    iv) Severe (3)
    v) Cannot assess

f) 3rd ventricle
    i) None (0)
    ii) Mild (1)
    iii) Moderate (2)
    iv) Severe (3)
    v) Cannot assess

g) 4th ventricle
    i) None (0)
    ii) Mild (1)
    iii) Moderate (2)
    iv) Severe (3)
    v) Cannot assess

7) Corpus Callosum

a) Is there atrophy in the corpus callosum region?
    i) Yes
    ii) No

b) Genu
    i) None (0)
    ii) Mild (1)
    iii) Moderate (2)
    iv) Severe (3)

- v) Cannot assess
- c) Body
  - i) None (0)
  - ii) Mild (1)
  - iii) Moderate (2)
  - iv) Severe (3)
  - v) Cannot assess
- d) Splenium
  - i) None (0)
  - ii) Mild (1)
  - iii) Moderate (2)
  - iv) Severe (3)
  - v) Cannot assess

8) Dementia diagnosis based on impression
   - a) Alzheimer’s Disease Dementia
   - b) Non-Alzheimer’s Disease Dementia

VII. Diagnostic criterion by cohort

Different dementia subtypes have different diagnostic guidelines and different centers may even have different criteria for inclusion in cohort studies. Given this variation, we herein provide summaries of such criteria for each of the datasets used for model development in our work. The information below is derived either directly from the documentation provided on each study's home webpage, or from highly cited articles that outline the requisite information.

- **ADNI**
  - Subjects for ADNI were classified as NC, MCI, or mild AD. With respect to diagnostic criteria, NC subjects had no memory complaints while MCI and AD subjects both had to have had complaints. Participants undergo a full review of past medical history, medications, and provide blood for APOE DNA testing, and undergo a battery of neuropsychological tests at baseline. All subjects also undergo 1.5T MRI, with additional subsets selected for 3T MRI, PET, and lumbar puncture. Criteria for clinical classification of NC MCI and AD are as follows:

    - NC:

      ➪ No Memory Complaints aside from those common to other normal subjects of that age range.

      ➪ Normal memory function documented by scoring at specific cutoffs on the Logical Memory II subscale (delayed Paragraph Recall) from the Wechsler Memory Scaled - Revised (the maximum score is 25):
      a) greater than or equal to 9 for 16 or more years of education
      b) greater than or equal to 5 for 8-15 years of education
      c) greater than or equal to 3 for 0-7 years of education.

      ➪ Mini-Mental State Exam score between 24 and 30 (inclusive) (Exceptions may be made for subjects with less than 8 years of education at the discretion of the project director).

      ➪ Clinical Dementia Rating = 0. Memory Box score must be 0.

      ➪ Cognitively normal, based on an absence of significant impairment in cognitive functions or activities of daily living
    - MCI:

      ➪ Memory complaint by subject or study partner that is verified by a study partner.

      ➪ Abnormal memory function documented by scoring below the education adjusted cutoff on the Logical Memory II subscale (Delayed Paragraph Recall) from the Wechsler Memory Scale – Revised (the maximum score is 25):

a) less than or equal to 8 for 16 or more years of education
b) less than or equal to 4 for 8-15 years of education
c) less than or equal to 2 for 0-7 years of education.

➪ Mini-Mental State Exam score between 24 and 30 (inclusive) (Exceptions may be made for subjects with less than 8 years of education at the discretion of the project director).

➪ Clinical Dementia Rating = 0.5. Memory Box score must be at least 0.5.

➪ General cognition and functional performance sufficiently preserved such that a diagnosis of Alzheimer's disease cannot be made by the site physician at the time of the screening visit.

MMSE between 24-30, memory complaint, objective memory loss measured by education-adjusted score on Wechsler Memory Scale Logical Memory II, CDR 0.5, preserved activities of daily living, absent dementia, and absence of significant cognitive impairment in other domains.

- AD:

  ➪ Memory complaint by subject or study partner that is verified by a study partner.

  ➪ Abnormal memory function documented by scoring below the education adjusted cutoff on the Logical Memory II subscale (Delayed Paragraph Recall) from the Wechsler Memory Scale – Revised (the maximum score is 25):
  a) less than or equal to 8 for 16 or more years of education
  b) less than or equal to 4 for 8-15 years of education
  c) less than or equal to 2 for 0-7 years of education.

  ➪ MMSE between 20 and 26 (inclusive) (Exceptions may be made for subjects with less than 8 years of education at the discretion of the protocol PI).

  ➪ Clinical Dementia Rating = 0.5, 1.0

  ➪ NINCDS/ADRDA criteria for probable AD

- **NACC:**
  - NACC employs a longitudinal data collection protocol using prospective, standardized clinical evaluation of subjects in the National Institute of Aging's Alzheimer's Disease Research Centers (ADRCs). Each center enrolls participants according to its own protocol. Subjects, along with their family and friends, if necessary, participate in annual screening questionnaires comprising NACC's Uniform Data Set (UDS) standards. Questionnaire results include neuropsychological testing, medical history, and present symptoms. Final

diagnosis is made by either a consensus team of experts, or the examining physician, with the exact arbiter varying according to the specific ADRC.

- **PPMI**
  - PPMI subjects are recruited at disease threshold, within two years of the time of first diagnosis by a treating clinician. While diagnostic criteria per se are not set forth in the study's documentation, participation for patients with Parkinson's disease (PD) is standardized by certain inclusion criteria, which include:
    - At least two of: bradykinesia, resting tremor, and rigidity OR either asymmetric resting tremor OR asymmetric bradykinesia.
    - No prior treatment for PD
    - Note expected to require PD medication within at least 6 months for baseline
    - At least 30 years old at the time of PD diagnosis
    - Hoehn and Yahr stage I or II at baseline
    - Dopamine transporter deficit per SPECT or VMAT deficit per VMAT-2 PET scan
    - Asymmetric resting tremor or asymmetric bradykinesia

- **LBDSU**
  - Diagnosis of Lewy Body dementia from the Lewy Body Dementia Research Center of Excellence at Stanford University follows diagnostic criteria set forth by the consensus of the Dementia with Lewy Bodies Consortium. While a full accounting of Core Clinical Features, Supportive Clinical Features, Indicative Biomarkers, and Supportive Biomarkers may be found elsewhere,[9] these guidelines define criteria for diagnosis of both probable and possible cases of Lewy Body dementia. In the present study, we utilized information from subjects meeting criteria for probably Lewy Body dementia, which is defined as either a) ≥2 core clinical features of DLB present with/without presence of indicative of biomarkers or b) only one core clinical feature present but with one or more indicative biomarkers.[9]
- **AIBL**
  - All volunteers in the AIBL study underwent a screening interview, comprehensive cognitive testing, health, and lifestyle questionnaires. Allocation of individuals to one of three diagnostic groups (NC, MCI, AD) was performed by a clinical review panel's consensus, which assessed both patients with a known history of MCI or AD diagnosis, as well as those recruited as NC who demonstrated any of the following conditions:
    - MMSE < 28/30
    - Failure on Logical Memory test
    - Clinical Dementia Rating (CDR) score ≥ 0.5

        - Medical history suggestive of the presence of illness likely to impair cognitive function
        - Informant or personal history suggestive of cognitive impairment
        - Consumption of medications or other substances that could affect cognition
        - Other evidence of significant cognitive difficulty on neuropsychological testing both
    - The clinical review panel consisted of two geriatric psychiatrists, a neurologist, a geriatrician, and five neuropsychologists. AD diagnoses included DSM-IV diagnostic criteria, ICD-10 dementia severity rating, and NINCDS-ADRDA diagnostic criteria. MCI diagnoses were made according to criteria set forth in Winblad[10] and Petersen,[11] and those presenting with previously diagnosed MCI were required to demonstrate a score of 1.5 standard deviations or more below age-adjusted mean on at least one neuropsychological test. Those reporting as NC were eligible for a diagnosis of MCI if they demonstrated performance at least 1.5 standard deviations on two or more neuropsychological tests in addition to having reported memory difficulties. In
- **OASIS**
    - The OASIS dataset includes participants enrolled into several ongoing studies through the Charles F. and Joanne Knight ADRC at Washington University in St. Louis. As in NACC, participants completed clinical assessments according to the UDS. Using the UDS, dementia status was assessed using the CDR score, with 0 indicating NC, 0.5 very mild impairment, 1 MCI, and 2 moderate dementia. Diagnostic impressions are also provided as a separate variable within this dataset by examining physicians and are available in separate data fields from the UDS-derived score.
- **FHS**
    - The Original Cohort of FHS participants underwent the Kaplan-Albert neuropsychological test battery in their fourteenth examination cycle and has since been monitored at regular intervals. Persons scoring below education-based cutoffs on MMSE or those who experience a decrease of at least three points between examinations are flagged for additional rounds of neurological and neuropsychological assessment. Family- or self-reported memory loss symptoms, as well as referral from FHS physicians and staff may similarly lead participants to in-depth cognitive assessments. All followup testing that remains concerning for dementia is assessed by a panel consisting of at least one neurologist and one neuropsychologist, who utilize available neurological/neuropsychological testing, a structured telephone interview with family members or caregivers, past FHS and medical history, as well as imaging and autopsy results where available. Dementia and MCI are diagnosed according to DSM criteria, and AD specifically is

diagnosed according to criteria from the National Institute of Neurological and Communicative Disorders and Stroke-Alzheimer's Disease and Related Disorders Association.[12]

- **NIFD:**
  - The Frontotemporal Lobe Dementia Neuroimaging Initiative encompasses individuals diagnosed with behavioral variant Frontotemporal Dementia (bvFTD), semantic variant Primary Progressive Aphasia (svPPA), and nonfluent variant Primary Progressive Aphasia, as well as age-matched controls for each of those cohorts. Recruited patients are drawn from clinical sites at the University of California, San Francisco, Massachusetts General Hospital, and the Mayo Clinic of Minnesota. bvFTD diagnostic criteria are set forth in the recommendations of the International Behavioral Variant FTD Criteria Consortium,[13] and criteria for diagnosis of primary progressive aphasia are similarly based upon international expert consensus which encompass clinical, imaging, and biomarker data.[14]

References:

1 Folstein, M. F., Folstein, S. E. & McHugh, P. R. "Mini-mental state": a practical method for grading the cognitive state of patients for the clinician. Journal of psychiatric research 12, 189-198 (1975).

2 Wechsler, D. San Antonio, Texas: The Psychological Corporation; 1987. Wechsler Memory Scale (Revised Manual)[Google Scholar].

3 Kaplan, E., Goodglass, H. & Weintraub, S. Boston naming test. (2001).

4 Reitan, R. M. Validity of the Trail Making Test as an indicator of organic brain damage. Perceptual and motor skills 8, 271-276 (1958).

5 Wechsler, D. WAIS-R: Wechsler adult intelligence scale-revised. (Psychological Corporation [and] Harcourt Brace Jovanovich, 1981).

6 Pfeffer, R. I., Kurosaki, T. T., Harrah Jr, C., Chance, J. M. & Filos, S. Measurement of functional activities in older adults in the community. Journal of gerontology 37, 323-329 (1982).

7 Sheikh, J. I. & Yesavage, J. A. Geriatric Depression Scale (GDS): recent evidence and development of a shorter version. Clinical Gerontologist: The Journal of Aging and Mental Health (1986).

8 Kaufer, D. I. et al. Validation of the NPI-Q, a brief clinical form of the Neuropsychiatric Inventory. The Journal of neuropsychiatry and clinical neurosciences 12, 233-239 (2000).

9 McKeith, I. G. et al. Diagnosis and management of dementia with Lewy bodies: Fourth consensus report of the DLB Consortium. Neurology 89, 88-100 (2017).

10 Winblad, B. et al. Mild cognitive impairment–beyond controversies, towards a consensus: report of the International Working Group on Mild Cognitive Impairment. Journal of internal medicine 256, 240-246 (2004).

11 Petersen, R. C. et al. Mild cognitive impairment: clinical characterization and outcome. Archives of neurology 56, 303-308 (1999).

12 Chêne, G. et al. Gender and incidence of dementia in the Framingham Heart Study from mid-adult life. Alzheimer's & Dementia 11, 310-320 (2015).

13 Rascovsky, K. et al. Sensitivity of revised diagnostic criteria for the behavioural variant of frontotemporal dementia. Brain 134, 2456-2477 (2011).
14 Gorno-Tempini, M. L. et al. Classification of primary progressive aphasia and its variants. Neurology 76, 1006-1014 (2011).
