# Supplementary material for: Multimodal deep learning for Alzheimer’s disease dementia assessment
Source: Nat Commun. 2022 Jun 20;13:3404. doi: 10.1038/s41467-022-31037-5 (PMC9209452; doi:10.1038/s41467-022-31037-5)
Supplement: Supplementary file 3 — Reporting Summary [file 41467_2022_31037_MOESM3_ESM.pdf]

nature portfolio

Corresponding author(s): Vijaya B. Kolachalama, PhD

Last updated by author(s): Apr 4, 2022

Reporting Summary

Nature Portfolio wishes to improve the reproducibility of the work that we publish. This form provides structure for consistency and transparency in reporting. For further information on Nature Portfolio policies, see our Editorial Policies and the Editorial Policy Checklist.

Statistics

For all statistical analyses, confirm that the following items are present in the figure legend, table legend, main text, or Methods section.

n/a	Confirmed	
☐	☒	The exact sample size (*n*) for each experimental group/condition, given as a discrete number and unit of measurement
☐	☒	A statement on whether measurements were taken from distinct samples or whether the same sample was measured repeatedly
☐	☒	The statistical test(s) used AND whether they are one- or two-sided<br>*Only common tests should be described solely by name; describe more complex techniques in the Methods section.*
☐	☒	A description of all covariates tested
☐	☒	A description of any assumptions or corrections, such as tests of normality and adjustment for multiple comparisons
☐	☒	A full description of the statistical parameters including central tendency (e.g. means) or other basic estimates (e.g. regression coefficient) AND variation (e.g. standard deviation) or associated estimates of uncertainty (e.g. confidence intervals)
☐	☒	For null hypothesis testing, the test statistic (e.g. *F*, *t*, *r*) with confidence intervals, effect sizes, degrees of freedom and *P* value noted<br>*Give P values as exact values whenever suitable.*
☒	☐	For Bayesian analysis, information on the choice of priors and Markov chain Monte Carlo settings
☒	☐	For hierarchical and complex designs, identification of the appropriate level for tests and full reporting of outcomes
☐	☒	Estimates of effect sizes (e.g. Cohen's *d*, Pearson's *r*), indicating how they were calculated

Our web collection on statistics for biologists contains articles on many of the points above.

Software and code

Policy information about availability of computer code

Data collection	All clinicians reviewed MRIs using 3D Slicer (version 4.10.2). Additionally, we used SAS (version 9.4) for statistical analysis and REDCap (version 11.1.3) for generating the questionnaire for clinicians. MRI pre-processing was performed using functions (e.g. BET) from the FSL (version 6.0) library.
Data analysis	Python (version 3.7.7) was used for software development. Each machine learning model was developed using PyTorch (version 1.5.1) and plots were generated using the Python library matplotlib (version 3.1.1) and numpy (version 1.187.1) was used for vectorized numerical computation. Other Python libraries used to support data analysis and visualization (e.g., tSNE) included pandas (version 1.0.3), scipy (version 1.3.1), tensorflow (version 1.14.0), tensorboardX (version 1.9), torchvision (version 0.6), and scikit-learn (version 0.22.1).

For manuscripts utilizing custom algorithms or software that are central to the research but not yet described in published literature, software must be made available to editors and reviewers. We strongly encourage code deposition in a community repository (e.g. GitHub). See the Nature Portfolio guidelines for submitting code & software for further information.

Data

Policy information about availability of data

All manuscripts must include a data availability statement. This statement should provide the following information, where applicable:

- Accession codes, unique identifiers, or web links for publicly available datasets
- A description of any restrictions on data availability
- For clinical datasets or third party data, please ensure that the statement adheres to our policy

This study includes data from eight cohorts. The studies are the Alzheimer's Disease Neuroimaging Intiative (ADNI: http://adni.loni.usc.edu/), the National Alzheimer's Coordinating Center (NACC: https://naccdata.org/), the frontotemporal lobar degenerative neuroimaging initiative (NIFD: https://ida.loni.usc.edu/

collaboration/access/appLicense.jsp;jsessionid=128F3A07C066CD297A62284C9A990001), the Parkinson's Progression Markerr Initiative (PPMI, https://www.ppmi-info.org/), the Austalian Imaging, Biomarker, and Lifestyle Flagship Study of Ageing (AIBL: https://aibl.csiro.au/adni/index.html), the Open Access Series of Imaging Studies (OASIS: https://www.oasis-brains.org/), the Framingham Heart Study (https://framinghamheartstudy.org/), and in-house data from the Lewy Body Dementia Center for Excellence at Stanford University (LBSDU: https://med.stanfrod.edu/poston-lab/LBD.html). Data from ADNI, AIBL, NACC, NIFD, OASIS and PPMI can be downloaded from publicly available resources. Data from FHS and LBDSU are available upon request and will be subjected to institutional approval.
Source data for figures are provided with this paper

Field-specific reporting

Please select the one below that is the best fit for your research. If you are not sure, read the appropriate sections before making your selection.

☒ Life sciences ☐ Behavioural & social sciences ☐ Ecological, evolutionary & environmental sciences

For a reference copy of the document with all sections, see nature.com/documents/nr-reporting-summary-flat.pdf

Life sciences study design

All studies must disclose on these points even when the disclosure is negative.

Sample size	Sample sizes were based on the availability of cases from the 8 cohorts mentioned previously in the Data section.
Data exclusions	Data were excluded from subjects who did not have at least one T1-weighted volumetric MRI scan within 6 months of an officially documented diagnosis. Additionally, we excluded all MRI scans with fewer than 60 slices. For subjects with multiple MRIs and diagnosis records within a 6 month period, we selected the closest pairing of neuroimaging and diagnostic label.
Replication	Consistent random seeds were set for all models in order to establish reproducibility of all analyses, which were repeated at regular intervals throughout the process of preparing and revising all results. The data from the ADNI, NACC, NIFD, PPMI, and OASIS studies are open access and the results can be replicated by following the methods described in the manuscript or by running the codes available in our GitHub repository. Some of the data from FHS and the entire LBDSU dataset require additional data requests to access restricted in-house datasets for replication of findings from these studies.
Randomization	When building the deep learning model, cases were randomized into training and validation groups under specific guidelines (described at length in the Methods and Supplementary Figures). When comparing performance of clinicians versus deep learning models, cases tested were randomly sampled from the cohorts (adjusting for age, gender, and education).
Blinding	In the comparison of clinicians versus deep learning model performance, clinicians were blinded to the documented clinical diagnoses of the cases presented.

Reporting for specific materials, systems and methods

We require information from authors about some types of materials, experimental systems and methods used in many studies. Here, indicate whether each material, system or method listed is relevant to your study. If you are not sure if a list item applies to your research, read the appropriate section before selecting a response.

Materials & experimental systems

n/a	Involved in the study	
☒	☐	Antibodies
☒	☐	Eukaryotic cell lines
☒	☐	Palaeontology and archaeology
☒	☐	Animals and other organisms
☒	☐	Human research participants
☒	☐	Clinical data
☒	☐	Dual use research of concern

Methods

n/a	Involved in the study	
☒	☐	ChIP-seq
☒	☐	Flow cytometry
☐	☒	MRI-based neuroimaging

Magnetic resonance imaging

Experimental design

Design type	Resting state structural MRI
Design specifications	For each cohort, we used all subjects, assuming the availability of at least one T1-weighted MRI scan taken within 6 months before or after the time of a clinical diagnosis. If a subject undertook multiple MRI scans and clinical diagnoses, we selected the closest pair of MRI and diagnosis, then included this pair of data only if the time span between the MRI date and diagnosis date was within 6 months. Because of this inclusion strategy, only 1 MRI scan per subject was used

for this study.

Behavioral performance measures: No behavioral performance measures were necessary for structural MRIs used in this study.

Acquisition

Imaging type(s): Structural

Field strength: 1.5 or 3 Tesla

Sequence & imaging parameters: Only T1-weighted MRI from all cohorts with acquisition type equal to 3D were used in this study without specifying other sequence parameters. The most common pulse sequence type of the MRIs from NACC is gradient recalled (GR), and only a few have research mode as the sequence type. Statistics of the following sequence parameters were estimated using 1000 randomly sampled MRIs: echo team (mean value: 0.0035 seconds, standard error: 0.0032 seconds); repetition time (mean value: 0.0133 seconds, standard error: 0.0106 seconds); flip angle (mean value: 19.05; standard error: 18.37).

Area of acquisition: Whole brain

Diffusion MRI ☐ Used ☒ Not used

Preprocessing

Preprocessing software: We used FMRIB Software Library v6.0 (FSL) (Analysis Group, Oxford University) for all preprocessing steps, including the following tools within that package: fslreorient2std (standard axis reorientation), robustfov (automated region-of-interest identification for robust registration), fslmaths (application of matrix operations), BET (brain extraction), FLIRT (linear registration), FAST (bias field correction), and FNIRT (nonlinear registration/segmentation)

Normalization: MRI scans from all datasets were preprocessed using a common pipeline implemented in FSL v6.0. Raw MRIs were first reoriented to a standard axis layout (using "fslreorient2std" tool) and then aligned to the MNI-152 template using a linear registration tools ("FLIRT") and automatically-identified region-of-interest ("robustfov" tools). These aligned MRIs were then skull-stripped (using "BET") and the resultant brains then underwent a second linear registration for fine-tuning of MNI alignments, as well as bias field correction for magnetic field inhomogeneities using the "FAST" tool. Finally, specific brain regions were segmented by aligning the Hammersmith adult brain atlas to registered brains using a non-linear registration ("FNIRT" tool).

Normalization template: Subjects were standardized to MNI-152 group space and parcellation of specific brain regions was performed according to the Hammersmith adult brain atlas (n30r95_SPM12_20170315), as described above.

Noise and artifact removal: We removed bias field inhomogeneities using the "FAST" tool within FSL.

Volume censoring: No volume censoring was used in this study.

Statistical modeling & inference

Model type and settings: We utilized a multimodal deep learning model for differential diagnosis, whose architecture and parameterr sharing strategy may be found described within the Methods section of our manuscript. We additionally tested CatBoost, XGBoost, Random Forest, Decision Tree, Support Vector Machine, K-Nearest Neighbor, and Multilayer Perceptron algorithms for comparison to our deep learning model.

Effect(s) tested: Task- and stimulus-related effects were not tested in this structural MRI study; rather, MRI scans were taken as clinical diagnostic tools and disease status was the outcome variable.

Specify type of analysis: ☐ Whole brain ☐ ROI-based ☒ Both

Anatomical location(s): A probabilistic atlas (Hammersmith adult brain atlas (n30r95_SPM12_20170315)) was used in order to determine anatomic regions of interest within the brain.

Statistic type for inference (See Eklund et al. 2016): We used voxel-wise SHAP values for statistical inference.

Correction: In the case of ANOVA testing for SHAP values in differing categories of neuropathologic severity, we followed significant inter-group differences with the Tukey honest significant difference test, which specifically accounts for multiple comparisons within its design. We do not report additional statistical methods requiring corrections.

Models & analysis

n/a	Involved in the study	
☒	☐	Functional and/or effective connectivity
☐	☒	Graph analysis
☐	☒	Multivariate modeling or predictive analysis

Graph analysis

We constructed weighted, acyclic graphs at the group levels, where each edge strength corresponded to the strength of association (measured by Pearson and Spearman's correlations) and each node radius was a weighted function of that node's degree. Though graphs were not used for inference purposes, they did provide visual evidence of differences in our dependent variable of AD status.

Multivariate modeling and predictive analysis

Our main predictive analysis of disease status proceeded via our multimodal deep learning framework, which includes both neuroimaging (MRI) and clinical variables obtained during dementia diagnostic workup. The details of our approach (including architecture and performance metrics) may be found in the Methods section of our main manuscript. We additionally reported the results of numerous "traditional" machine learning classifiers which use various subsets of deep learning-derived neuroimaging features and standard clinical information. We summarized model performance using area under Receiver-Operating Characteristic curve (AUC) and Precision-Recall (PR) curves within the main manuscript. ASlso, accuracy, F1-score, sensitivity, specificity, and Matthew's Correlation Coefficient (MCC) values for various classifiers tested in our manuscript may be found within our supplementary materials.
